# Establishment and validation of a plasma oncofetal chondroitin sulfated proteoglycan for pan-cancer detection

**DOI:** 10.1038/s41467-023-36374-7

**Published:** 2023-02-06

**Authors:** Pei-Fen Zhang, Zi-Yi Wu, Wen-Bin Zhang, Yong-Qiao He, Kexin Chen, Tong-Min Wang, Haixin Li, Hong Zheng, Dan-Hua Li, Da-Wei Yang, Ting Zhou, Chang-Mi Deng, Ying Liao, Wen-Qiong Xue, Lian-Jing Cao, Xi-Zhao Li, Jiang-Bo Zhang, Si-Qi Dong, Fang Wang, Mei-Qi Zheng, Wen-Li Zhang, Jianbing Mu, Wei-Hua Jia

**Affiliations:** 1 State Key Laboratory of Oncology in South China, Collaborative Innovation Center for Cancer Medicine, Sun Yat-sen University Cancer Center, Guangzhou, 510060 China; 2 Department of Radiation Oncology, Fujian Medical University Cancer Hospital, Fujian Cancer Hospital, Fuzhou, 350014 China; 3 Department of Epidemiology and Biostatistics, National Clinical Research Center for Cancer, Key Laboratory of Molecular Cancer Epidemiology of Tianjin, Tianjin Medical University Cancer Institute and Hospital, Tianjin, 300060 China; 4 Department of Cancer Biobank, Key Laboratory of Cancer Prevention and Therapy of Tianjin, Tianjin’s Clinical Research Center for Cancer, National Clinical Research Centre of Cancer, Tianjin Medical University Cancer Institute and Hospital, Tianjin, 300060 China; 5 School of Public Health, Sun Yat-sen University, Guangzhou, 510060 China; 6 Laboratory of Malaria and Vector Research, National Institute of Allergy and Infectious Diseases, National Institutes of Health, Rockville, MD USA

**Keywords:** Diagnostic markers, Tumour biomarkers, Tumour biomarkers

## Abstract

Various biomarkers targeting cell-free DNA (cfDNA) and circulating proteins have been tested for pan-cancer detection. Oncofetal chondroitin sulfate (ofCS), which distinctively modifies proteoglycans (PGs) of most cancer cells and binds specifically to the recombinant *Plasmodium falciparum* VAR2CSA proteins (rVAR2), is explored for its potential as a plasma biomarker in pan-cancer detection. To quantitate the plasma ofCS/ofCSPGs, we optimized an ELISA using different capture/detection pairs (rVAR2/anti-CD44, -SDC1, and -CSPG4) in a case-control study with six cancer types. We show that the plasma levels of ofCS/ofCSPGs are significantly higher in cancer patients (*P* values, 1.2 × 10^−2^ to 4.4 × 10^−10^). Validation studies are performed with two independent cohorts covering 11 malignant tumors. The individuals in the top decile of ofCS-CD44 have more than 27-fold cancer risk (OR = 27.8, 95%CI = 18.8–41.4, *P* = 2.72 × 10^−62^) compared with the lowest 20%. Moreover, the elevated plasma ofCS-CD44 could be detected at the early stage of pan-cancer with strong dose-dependent odds risk prediction.

## Abstract (web summary)

Developing multi-cancer early detection (MCED) tests based on cfDNA or protein markers holds great promise for effective cancer screening. Here, the authors show a sensitive, non-invasive pan-cancer detection method that concurrently detects the oncofetal chondroitin sulfate (ofCS) glycans and its protein backbone in plasma.

## Introduction

Early detection of cancers remains one of the most promising approaches to improving long-term survival and reducing cancer patients’ mortality. Currently, available cancer screening biomarkers are mainly utilized to detect a particular cancer type (single organ screening) with limited sensitivity and specificity^1^. In current guidelines, just four cancers—breast, cervix, colorectum, and prostate, were recommended for average-risk population screening by American Cancer Society^2,3^. Therefore, the development of tools for universal or multi-organ cancers (including the less common ones) screening has gained significant attention in recent years.

Universal cancer screening (UCS)^4^, which is based on combinations of circulating biomarkers such as tumor antigens (associated glycans), cancer-derived circulating-free DNA (cfDNA), RNA, and exosomes, was tested recently and showed great potential to revolutionize pan-cancer early detection, including cancers with no available screening modalities^5,6^. In their large prospective study (DETECT-A), Lennon et al. ^5^ applied a multi-analyte panel consisting of 16 ctDNA and nine circulating protein markers, referred to as CancerSEEK^7^, to screen a cohort of 10,006 women. During the 12-month follow-up period, 96 cases of cancer were detected, of which CancerSEEK detected 26 cancers in ten different organs. The Circulating Cell-free Genome Atlas (CCGA) study detected and localized more than 50 cancer types using cfDNA methylation signatures combined with the machine-learning approach^6^. The multi-cancer early detection (MCED) test performance in this study varies from 39 to 92% based on cancer stage and is further validated using an independent, large-scale clinical substudy^8^. A similar report by Zhang *et al*. characterized the detectability and molecular feature of ctDNA, particularly the mutational landscape of pan-cancer ctDNA, across different cancer types in over 10,000 Chinese patients^9^. Other molecules such as platelet RNA^10^, KRT8^11^, and exosomes^12^ were identified recently as potential pan-cancer biomarkers through the multi-scale integrated analysis, but they are also inadequate for detecting certain cancer types. Nevertheless, further refinement of these tests might be actualized with additional robust biomarkers that are broadly expressed across tumor types with site-prediction potential and could be detected non-invasively on a single medium (e.g., blood, urine, saliva, or other)^13^. It has been evidenced that the diagnostic and prognostic performance of existing serological biomarkers, mostly glycoproteins, could be significantly improved when simultaneously measuring the glycans in combination with the protein backbone^14–16^. For example, the serum level of fucosylated α-fetoprotein (AFP), but not AFP alone, allows successful discrimination between hepatocellular carcinoma (HCC) and benign live diseases^17^. Similarly, a clinical biomarker for prostate cancer, PSA, could positively detect benign prostatic hyperplasia and prostatitis, while the percentages of α2.3-sialic acid and core-fucosylated glycovariants in total serum PSA provide sufficient reliability in differentiating high-risk patients^18,19^. Furthermore, combined detection of plasma carbohydrate antigen19–9 (CA19-9)^20^, sTRA glycans^21^, or thrombospondin-2 (THBS2)^22^ provides better performance in pancreatic cancer diagnosis.

ofCS, a distinct chondroitin sulfate glycosaminoglycan epitope that is usually restricted to trophoblastic cells in the placenta, has been found in a large number of tumors, allowing for a broad targeting of human cancer cells^23^. This unique glycosaminoglycan (GAG) chain is covalently attached to multiple proteoglycans such as the cluster of differentiation 44 (CD44), chondroitin sulfate proteoglycan 4 (CSPG4), or Syndecan-1 (SDC1) in various cancer cells or secreted into the microenvironment surrounding the cells and bodily fluids including blood and urine^24–27^. Importantly, rVAR2, a recombinant fragment of *Plasmodium falciparum* VAR2CSA protein, has been evolutionarily selected for the parasites binding in the placenta through a specific chondroitin sulfate (CS) oligosaccharide motif^28–30^, showing an unprecedented high specificity and affinity for ofCS binding^23^. The binding of rVAR2 to cancer is nearly universal, independent of EMT processes and tumor origin, and highly specific with minimal to absent binding in normal tissue, except for the placenta^23,31,32^. As such, rVAR2 has been tested to retrieve circulating tumor cells from cancer patients’ blood^31^ or ofCS-modified proteoglycans (ofCSPGs) in bladder cancer patients’ urine^27^.

We, therefore, hypothesized that plasma ofCS or ofCSPGs might be a useful pan-cancer biomarker if a sensitive, specific detection approach can be established. To this end, we optimized an ELISA using different capture/detection pairs to concurrently detect the ofCS glycans and its protein backbone for plasma ofCS/ofCSPGs measurements. Antibodies targeting the backbone of ofCSPGs (anti-CD44, SDC1, and CSPG4) and rVAR2 containing variable ofCS glycans binding domains were tested. The optimized ELISA measuring ofCS glycan and ofCSPGs was then evaluated for pan-cancer detection in a discovery study and validated in two independent case-control study populations.

## Results

### rVAR2 with high, but variable, chondroitin sulfate A (CSA) binding affinity

**Fig. 1:**
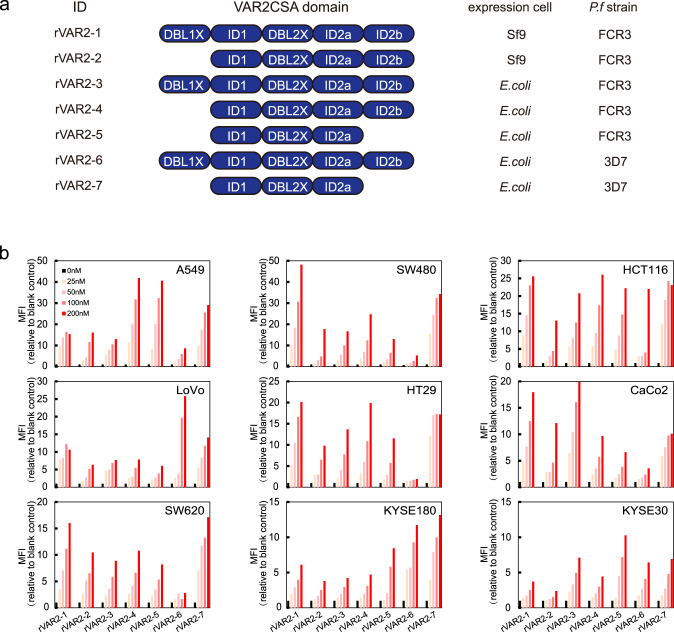
CSA binding ability of rVAR2. **a** Schematic illustration of rVAR2 with different binding domains and the expression cell of seven rVAR2 to CSA. ^*^ rVAR2-3 was used as the detection molecular at the modified ELISA test. **b** Relative mean fluorescence intensity (MFI) of lung adenocarcinoma cell line (A549), colorectal carcinoma cell lines (SW480, HCT116, LoVo, HT29, CaCo2, SW620), and esophageal cell carcinoma cell lines (KYSE180 and KYSE30). Cells were incubated with serial dilution of rVAR2 as indicated and detected by flow cytometry using anti-V5-FITC.

Previous studies suggested that the minimal CSA binding region in VAR2CSA is DBL2X-ID2b, with the need for DBL1X or DBL3X for full affinity binding^33–36^, and the mutations located inside these binding regions could dramatically affect the binding efficacy of VAR2CSA^37^. To obtain high-affinity ofCS binding rVAR2, we produced seven recombinant fragments of VAR2CSA proteins (rVAR2-1 to −7), containing variable CSA binding domains and sequence variants using *E.coli* or baculovirus expression system (Fig. 1a and Supplementary Fig. 1a–c). A previously reported recombinant fragment (ID1-DBL2X-ID2a)^23^ was used as the positive control. The short-form rVAR2 contains ID1-DBL2X-ID2a-ID2b domains, and the long-form rVAR2 includes an additional DBL1X domain (DBL1X-ID1-DBL2X-ID2a-ID2b). The sequence variants tested were harbored in a highly polymorphic VAR2CSA sequence between FCR3 and 3D7, two distinct laboratory *P. falciparum* strains to represent distinct “dimorphic” polymorphism of this gene.

The binding ability of all rVAR2 to CSA was determined by the real-time surface plasma resonance (SPR) technique (Biacore T200, GE), and the equilibrium dissociation constant K_D_ of these proteins were summarized in Supplementary Fig. 1d. The high binding affinity to CSA was confirmed for all rVAR2 at the nanomolar range of *K*_D_. All long-form rVAR2 (−1, −3, and −6) bind more efficiently than their short-form partner (rVAR2-2, −4, −5, and −7). The rVAR2 from the *E.coli* expression system generally have higher CSA binding affinity than those from the baculovirus expression system. The sequence variants between 3D7 and FCR3 appear also affect the CSA binding affinity, in which the rVAR2-6 (3D7 sequence) shows six times higher than the rVAR2-3 (FCR3 sequence). The high but variable binding affinity of all rVAR2 proteins offers an optimistic tool for further detecting its binding targets distributed on cancer cells or secreted inside the extracellular fluid (ECF) such as plasma.

### Binding of rVAR2 to multiple cancer cells

To evaluate the binding efficiency of all rVAR2 to cancer cells, we developed a flow cytometry assay relying on the interaction between V5-tagged rVAR2 and ofCS glycan expressed on cancer cells. Two blood samples from Leukemia patients and nine different cancer cell lines with variable origin, including lung adenocarcinoma cell line (A549), colorectal carcinoma cell lines (SW480, HCT116, LoVo, HT29, CaCo2, SW620), and esophageal cell carcinoma cell lines (KYSE180, KYSE30), were used to test the binding efficiency of all rVAR2. The successful bindings of rVAR2 to cancer cell lines were evidenced by significantly increased mean fluorescence intensity in a concentration-dependent manner (Fig. 1b). The binding efficacies of 7 rVAR2 to cancer cell lines are inconsistent with the above-mentioned CSA binding data (Supplementary Fig. 1d). Variable performances are found among different cell lines. rVAR2-1 and rVAR2-6, which contain two binding domains from FCR3 and 3D7, showed efficient binding in five cancer cell lines (SW480, HCT116, SW620, CaCo2, and HT29), and three cancer cell lines (LoVo, HCT116, and KYSE180), respectively, while the rVAR2-2 (one binding domain from FCR3) displayed overall poor performance than other rVAR2 (Fig. 1b), indicating the possible structural heterogenicity of ofCS glycan expressed on the cancer surface. Importantly, not only to cultured cancer cells, all rVAR2 were able to bind efficiently to blood cancer cells from Leukemia patients with no detectable binding to normal blood cells (Supplementary Fig. 1e).

To verify the location of ofCS in cancer cells, we performed immunofluorescence analysis with all rVAR2. Apparent staining, indicating the specific binding of rVAR2 with ofCS, was mainly found on cytoplasm and cancer cell surfaces, and the representative confocal microscopy images were shown in Supplementary Fig. 2a. Consistent with the aforementioned FACS data, the immunofluorescence signals are varied among different cancer cell lines.

### rVAR2 modified sandwich ELISA to detect plasma ofCS/ofCSPG in cancer patients

**Fig. 2:**
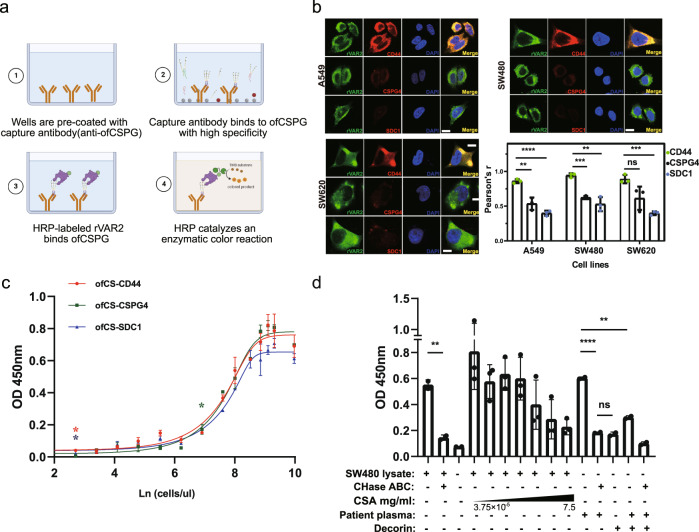
Schematic of sandwich ELISA, the sensitivity and specificity to detect ofCSPGs. **a** Schematic of sandwich ELISA workflow. The figure was created with Biorender.com. **b** Colocalization of ofCS with core proteins detected by rVAR2-3 and anti-CD44, CSPG4, and SDC1 antibodies (*n* = 3, biologically independent experiments). *P* = 0.0039 and *P* < 0.0001 for CD44 vs. CSPG4 and CD44 vs. SDC1 in A549, *P* = 0.0001 and *P* = 0.0025 for CD44 vs. CSPG4 and CD44 vs. SDC1 in SW480, *P* = 0.0549 and *P* = 0.0002 for CD44 vs. CSPG4 and CD44 vs. SDC1 in SW620. *R* = 1 represents perfect correlation. Scale bar **=** 10 μm. **c** The sensitivity of ELISA for ofCS-CD44, -CSPG4, and -SDC1 biomarkers. The cancer cell line (SW480) spiked samples were tested. **d** The ELISA specificity was detected by the competitive assay with gradient dilution of CSA (3.75 × 10^−5^–7.5 mg/mL), decorin, and chondroitinase-treated cancer cell line or plasma samples (*n* = 3, 2, and 2, respectively, biologically independent experiments). The chondroitinase treatment of the cancer cell line and patient plasma sample significantly reduced the rVAR2 binding (*P* = 0.0062 and *P* < 0.0001 respectively). No significant rVAR2 binding was detected in the decorin spiking sample indicating specific capture of the anti-CD44 antibody. Student *t*-test, all the tests were two-sided, data were shown as mean ± SD, ns. no significant, *p value <0.05, **p value <0.01, ***p value <0.001, ****p value <0.0001).

The broad bindings of rVAR2 to variable cancer cells support the efforts of pan-cancer biomarker development with a simple, efficient detection method. Early evidence suggested that ofCS uniquely modify a vast array of glycoproteins, either located on the cancer cell surface or secreted into extracellular fluid (ECF), predominantly the plasma^38,39^. For example, the ofCS-modified proteoglycan such as ofCS-CD44, -CSPG4, and -SDC1 can present as free form, attached with CTC, or largely with cancer-derived exosomes in plasma. The colocalizations of the ofCS with their core proteins (CD44, CSPG4, and SDC1) were detected on various cancer cells by immunofluorescence analysis (Fig. 2b and Supplementary Fig. 2a, b). Variable levels of colocalization between ofCS and their core proteins were detected among different cancer cell lines. The ofCS-CD44 displayed a higher colocalization rate in seven out of nine cancer cell lines (Pearson’s correlation coefficient >0.5), compared with ofCS-SDC1 (three out of nine) and ofCS-CSPG4 (four out of nine). The no-completed colocalization of ofCS with their core proteins reflects the dynamic ofCS modification processing of chondroitin sulfate proteoglycans (CSPGs) in the cancer cell. To efficiently capture these plasma ofCS glycans, we optimized an ELISA assay with different capture and detection pairs (anti- CD44, -CSPG4, -SDC1, and rVAR2), and tested their performance of detection ofCSPGs in plasma of malignant tumor patients. The schematic of the sandwich ELISA workflow is shown in Fig. 2a. In brief, the anti-CSPGs antibodies were set as coating molecules to capture the individual proteoglycan, and the rVAR2-5 was aimed to capture the total ofCS that existed in the specimen. After the specific capture of targeted ofCS, the HRP-labeled rVAR2-3 was applied to quantify the amount of ofCS in plasma samples. Since no standard ofCS-CD44, ofCS-SDC1, and ofCS-CSPG4 are available, the sensitivity and specificity of the optimized ELISA were characterized by cancer cell line (SW480) spiked sample and CSA/decorin competitive assay including chondroitinase-treated cancer cell line or plasma samples (Fig. 2c, d). As shown in Fig. 2c, the sensitivity of ofCS-CD44, ofCS-SDC1, and ofCS-CSPG4 are found to be at the amount of ofCS-CSPGs in 15 (*P* = 0.0197), 15 (*P* = 0.0102), and 1000 (*P* = 0.0274) cells ul^−1^, respectively. The linear range of the test is from 15 to 7000 cells ul^−1^ for ofCS-CD44, ofCS-SDC1, and ofCS-CSPG4. In the competitive assay, gradient dilution of CSA (3.75 × 10^−5^−7.5 mg mL^−1^) was co-incubated with SW480 lysates before the addition of recombinant VAR2CSA (Fig. 2d). The binding of VAR2CSA to ofCS-CD44 decreased significantly along with the increase of CSA concentration. In addition, the binding of recombinant VAR2CSA to cancer cells (SW480), patient plasma, or decorin is abolished if the samples were treated with chondroitinase before binding analyses (Fig. 2d and Supplementary Fig. 2c), indicating the specific binding of recombinant VAR2CSA.

**Fig. 3:**
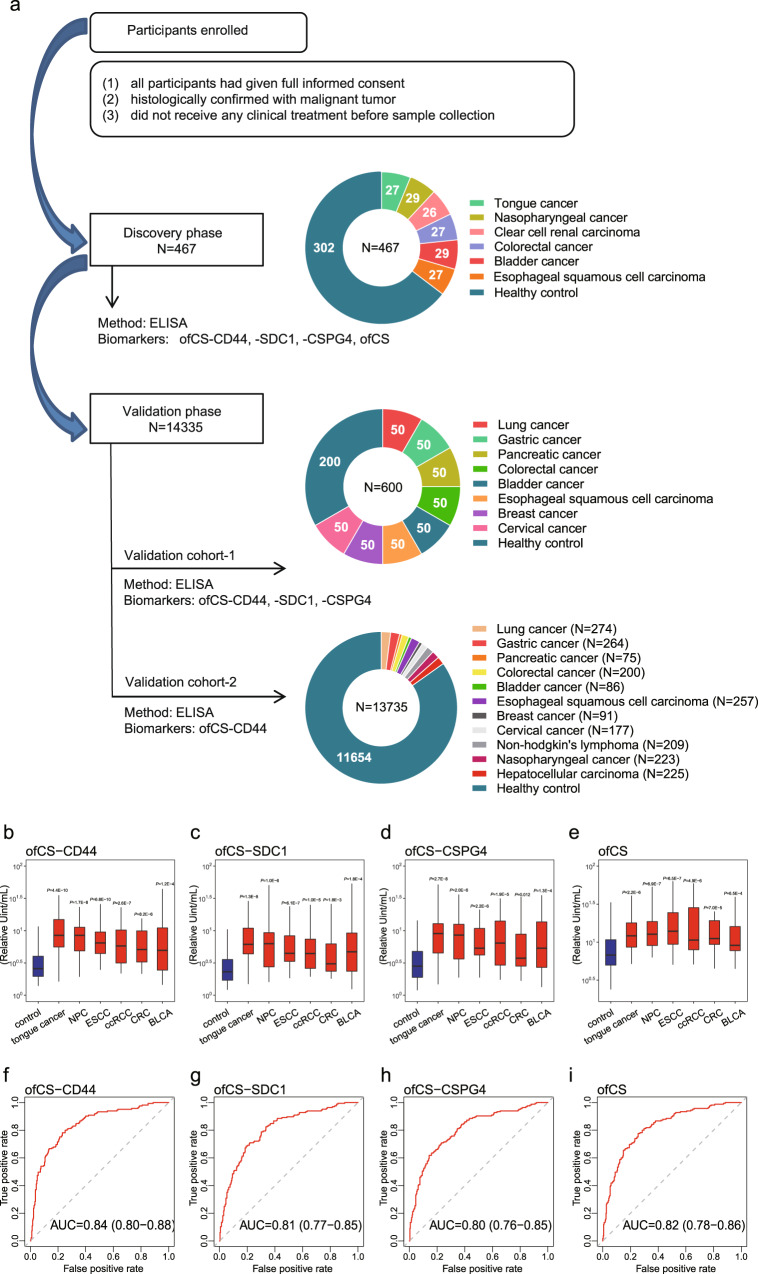
rVAR2 modified sandwich ELISA to detect plasma ofCS/ofCSPG in cancer patients at discovery study. **a** The study designed for the development and validation of a novel plasma oncofetal chondroitin sulfated proteoglycans for pan-cancer detection. **b**–**e** Plasma ofCS-CD44, -SDC1, -CSPG4 and total ofCS levels (median with inter-quartile range) from healthy individuals (*n* = 302) versus cancer patients with tongue cancer (*n* = 29), esophageal squamous cell carcinoma (ESCC, *n* = 27), nasopharyngeal carcinoma (NPC, *n* = 27), clear cell renal cell carcinoma (ccRCC, *n* = 27), colorectal cancer (CRC, *n* = 26), and bladder cancer (BLCA, *n* = 29), respectively. *P* for Mann–Whitney *U*-test. The middle line in the boxplot displays the median, and the box indicates the first and third quartiles. Value in each figure was compared with the first group. All the tests were two-sided. **f**–**i** ROC curves analysis for the diagnosis of malignant tumor patients (*n* = 165) from healthy individuals (*n* = 302) using the plasma ofCS, ofCS-CD44, -SDC1, and -CSPG4. The dotted diagonal line denotes an AUC of 0.50.

Using a biomarker discovery set consisting of 302 healthy controls and 165 malignant tumor patients (Fig. 3a), we found that the plasma ofCS-CD44, -SDC1, -CSPG4, and total ofCS in all types of cancer tested were significantly higher than that in healthy controls (Fig. 3b–e). The area under the receiver operating characteristic (ROC) curve (AUC) of ofCS-CD44, SDC1, CSPG4, and total ofCS was 0.84 (95% CI = 0.80−0.88), 0.81 (95% CI = 0.77−0.85), 0.80 (95% CI = 0.76−0.85), and 0.82 (95% CI = 0.78−0.86) (Fig. 3f–i). The diagnosis values of the ofCSPGs for individual cancer types were summarized in Supplementary Fig. 3a, implying that ofCS-CD44 can distinguish cancer better than other ofCSPGs. Interestingly, the total level of CD44 in plasma is not significantly different between cancer patients and controls (Supplementary Fig. 3c). It appears that only a proportion of CD44, not all, are modified by ofCS in cancer patients, which can be specifically detected by rVAR2.

To determine whether combined ofCSPGs could constitute a more discriminatory panel for cancer detection, we performed logistic regression to estimate the combined discriminative ability. With the combination of any of two or three and all the glycoproteins together, a slightly improved AUC was detected compared to the AUC of ofCS-CD44 alone (Supplementary Table 3).

### Validation of plasma ofCS modified CD44 marker in pan-cancer detection

**Fig. 4:**
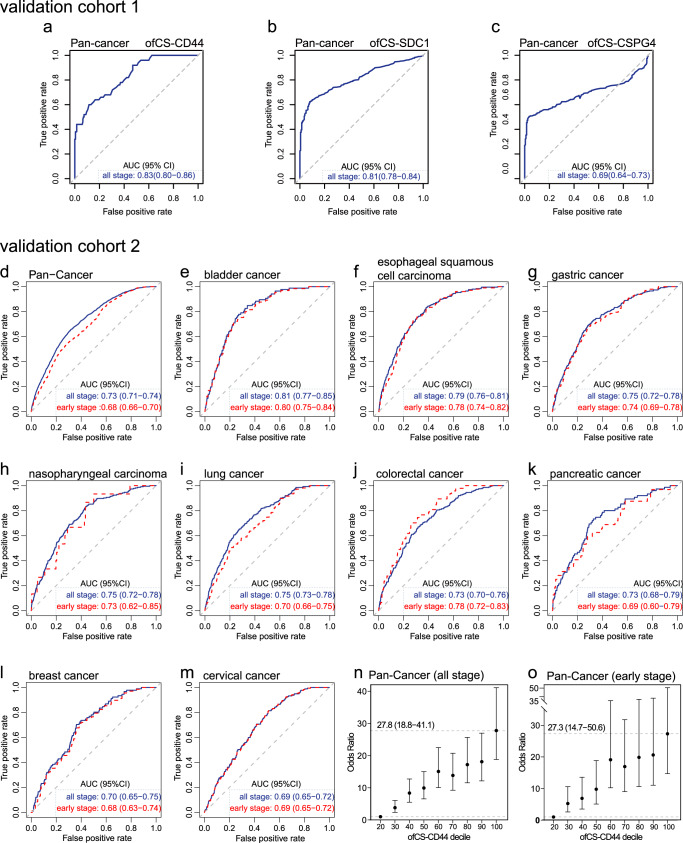
Validation of plasma ofCS modified CD44 in different types of the malignant tumor. **a**–**c** ROC curves analysis for the diagnosis of all stages of the pan-cancer set in validation study 1. **d** ROC curves analysis for the diagnosis of all stages (blue) of the pan-cancer set consisting of nine solid malignant tumor types except for hepatocellular carcinoma and early-stage patients (red) using the plasma ofCS-CD44. The dotted diagonal line denotes an AUC of 0.50. **e**–**m** ROC curves of all-stage (blue) and early-stage (red) bladder, esophageal squamous cell carcinoma, gastric, nasopharyngeal, lung, pancreatic, breast, and cervical cancer, respectively. **n**, **o** The OR and 95% CIs of the plasma ofCS-CD44 and cancer risk by decile at validation study for all stage patients of pan-cancer (*n* = 13,735) (**n**) and early-stage patients (*n* = 891) (**o**). Multiple logistic regression analysis was used to calculate the ORs adjusted for sex and age. All the tests were two-sided. The solid dots in the center for the error bars are the OR values, and the error bars are the corresponding 95% confidence intervals of the ORs. The dashed lines represent the OR values for samples with ofCS-CD44 ≥90% (upper line) and ofCS-CD44 <20% (lower line).

To validate the markers’ performance, we conducted two independent cohorts (Fig. 3a). The validation cohort 1 includes 400 cancers (eight different cancer types) and 200 controls. As shown in Fig. 4a–c, the AUC of ofCS-CD44, ofCS-CSPG4 and ofCS-SDC1 for pan-cancer detection is 0.83(95% CI: 0.80–0.86), 0.69 (95% CI: 0.64–0.73), and 0.81 (95% CI: 0.77–0.84), respectively. The comparable AUC with the discovery stage is detected for ofCS-CD44 and ofCS-SDC1 biomarkers, while AUC for ofCS-CSPG4 is decreased, possibly due to the higher background in ELISA assay (Supplementary Fig. 3b). The relatively higher ELISA variable of ofCS-SDC1 was detected in the validation cohort. Indeed, the Nagelkerke R^2^ analysis (an index of goodness of fit for the model) showed that ofCS-CD44 has the highest *R*^2^ in the discovery and validation cohort (0.29 and 0.43, respectively) compared with other markers (SDCI: 0.22 and 0.39; CSPG: 0.22 and 0.24, respectively). Considering the biomarker sensitivity, ofCS-CSPGs colocalization, and assay stability, we selected ofCS-CD44 for further validation of its’ pan-cancer detection performance in an extensive dataset (2081 cases and 11654 community-based healthy controls) with 11 cancer types, including bladder cancer, esophageal squamous cell carcinoma, gastric cancer, nasopharyngeal carcinoma, lung cancer, colorectal carcinoma, pancreatic cancer, breast cancer, cervical cancer, non-Hodgkin’s lymphoma, and hepatocellular carcinoma (Fig. 3a).

Consistent with the discovery and validation cohort 1 study, the plasma ofCS-CD44 performed well to distinguish malignant cancer, except for non-Hodgkin’s lymphoma and hepatocellular carcinoma. The AUC was 0.73 (95% CI = 0.71–0.74) for the pan-cancer detection (Fig. 4d) and varied among different cancer types (Fig. 4e–m and Supplementary Table 5). For example, the plasma ofCS-CD44 was more effective for bladder cancer detection, with an AUC of 0.81 for all stage patients. For gastrointestinal cancer, esophageal squamous cell carcinoma, gastric cancer, colorectal cancer, and pancreatic cancer, the AUC was 0.79 (95% CI = 0.76–0.81), 0.75 (95% CI = 0.72–0.78), 0.73 (95% CI = 0.70–0.76), and 0.73 (95% CI = 0.68–0.79), respectively. It was also well replicated in NPC with an AUC of 0.75 (95% CI = 0.72–0.78). Moreover, the elevated plasma ofCS-CD44 was significantly associated with increased odds of malignant disease. When comparing individuals in the lowest 20 percent versus the highest plasma ofCS-CD44 decile, there was an ~27-fold higher risk of malignant cancer (OR = 27.8, 95% CI = 18.8–41.1; *P* = 2.72 × 10^−62^, Fig. 4n). Considering the heterogeneity of malignant tumors, we separately analyzed the participant according to the cancer types (Supplementary Table 4). Interestingly, the clear plasma ofCS-CD44 amount-dependent risk for each kind of malignant tumor was detected, especially for colorectal cancer, nasopharyngeal carcinoma, esophageal squamous cell carcinoma, and cervical cancer, with a risk for the top decile ranging 8.9 to 25.5. This provides strong evidence that elevated plasma could be a useful factor in assessing cancer risk.

### Plasma ofCS modified CD44 is a promising biomarker for early detection

To investigate whether the plasma ofCS-CD44 could be a valuable biomarker for early-stage malignant tumor detection, we further assessed its performance in different cancer stages. The TNM stage I and TNM stage II were considered early stages in this study. As shown in Fig. 4d–m, the AUCs for early-stage cancer detection ranged from 0.68 to 0.80, which is similar to the all-stage cancer detection for the corresponding cancer type, indicating that plasma of CS-CD44 could be a promising biomarker for early detection. Furthermore, a strong association of the cancer risk with plasma ofCS-CD44 was observed not only in all-stage (Fig. 4n), but also in the early-stage patients, with increasing risk by plasma ofCS-CD44 decile, and the OR of malignant cancer for those in the top of CS-CD44 decile was 27.3 (95% CI = 14.7–50.6; *P* = 7.26 × 10^−26^, Fig. 4o). To check the pathological stage of the disease that influences the plasma concentration of ofCS, we analyzed the details of TNM staging of these 11 cancer types and their relationship with plasma ofCS-CD44. We found that plasma ofCS-modified CD44 level was significantly associated with progressive pathologic TNM stage in some cancers, such as breast cancer (BRCA), lung cancer (LC), and liver hepatocellular carcinoma (HCC) (Supplementary Fig. 4a–d), indicating its’ prognostic significance of cancer detection. However, further studies with larger sample sizes are needed to confirm these findings.

## Discussion

The conceptual basis for noninvasive, multi-organ cancer screening biomarkers is supported by strong epidemiological and biological evidence and the availability of many high-performance technologies^13^. The characteristic alterations of cancer cells are commonly detected in genetically, epigenetically altered DNA^40,41^, and qualitative and quantitative changes in various proteins and metabolite profiles^14,42,43^, some of which could serve as potentially valuable pan-cancer biomarkers. Indeed, several new multi-cancer early detection tests have been developed based on cfDNA/ctDNA methylation and fragmentation analysis^44–46^. For example, >300, 477, and >100,000 cancer-specific differentially methylated regions (DMRs) are targeted in cfMeDIP-seq, PanSeer, and Galleri, respectively, for multiple cancer detection.

Here, we evaluated seven sequence-varied rVAR2 for their detectability of the unique cancer-derived ofCS glycan or ofCSPGs, and established and validated the plasma ofCS-CD44 as a novel pan-cancer biomarker. As briefed in the introduction, the rationale for evaluating the rVAR2 based ofCS/ofCSPGs detection pan-cancer biomarker is as follows: (i) rVAR2 consists of core-binding domains of VAR2CSA, an evolutionarily selected *P. falciparum* protein for tight parasites binding to trophoblasts in placenta^47,48^. (ii) rVAR2 can specifically bind to 4-O-sulfated CS^31^, a re-expressed fetal antigen on many cell surface proteins from tumor samples^23^. (iii) rVAR2 can bind the various cancer cells independent of tumor origin^23^. (iv) rVAR2 has been successfully used to capture CTCs in blood^49^. (v) importantly, but not thoroughly tested, the effects of mutations (3D7 vs. FCR3) in rVAR2 core-binding domains on their binding capability to cancer cells. The sequence of the DBL2 and ID1 domains in VAR2CSA shows the distinct “dimorphic” structure, which divides the *P. falciparum* strains into two phylogenetic groups, representatively 3D7 and FCR3^50^. As reported in a recent study^37^, the variants in VAR2CSA can significantly impact the VAR2CSA protein binding to CSA. Here, we produced 7 different rVAR2 covered both dimorphic types using two in vitro protein expression systems – *E. coli vs. baculovirus*. High binding affinities, but varied dramatic (25 ×), of these rVAR2 to CSA were detected, laying a good foundation for pan-cancer detection. Multiple binding domains (DBL1X and DBL2X) in rVAR2, as well as the *E.coli* expression, could increase CSA binding. We further confirmed that all rVAR2 bind to ofCS in various cancer cell lines originating from both adenocarcinoma and squamous cell carcinoma in a concentration-dependent manner, agreed with previous reports^23^. However, the binding profiles of these rVAR2 to cancer cells are inconsistent with their binding to CSA. The rVAR2-1, a less CSA binding fragment, displayed a better binding ability to several cancer lines, while a similar property was shown for all rVAR2 in HCT116 cancer cell binding. This could be due to the heterogeneity ofCS glycan expressed in tumor cells, suggesting that the number and mutations in rVAR2 binding domains should be carefully considered when designing an ofCS-based pan-cancer detection biomarker.

In the efforts to develop a simple, efficient tool for pan-cancer detection, we optimized an ELISA assay to quantify the plasma ofCS/ofCSPG amounts in cancer patients. The plasma glycosaminoglycans possibly exist in various forms, such as the free form or attached to its core proteins on CTCs or exosomes^12,51–53^. Evidenced by improved performance of other serological cancer biomarkers, mostly single organ targeted, such as fucosylated α- AFP for HCC^17,54^, glycosylated PSA for prostate cancer^17^, and CA19-9/ THBS2 marker for pancreatic cancer^22^, simultaneous targeting ofCS with its modified core proteins were focused on to develop the pan-cancer biomarker in our study. Three ofCS modified glycoproteins (ofCS-CD44, SDC1, and CSPG4) detected across multiple cancer cells by rVAR2 were selected because of their important roles in cancer progression and metastasis^55,56^. CD44 is a multifunctional cell surface adhesion receptor highly expressed in many cancers^57^ and tumor-derived extracellular vesicles (TEVs)^58^. CSPG4 and SDC1 are also essential to cell surface adhesion molecule contributing to cancer progression by promoting cell proliferation, metastasis, invasion, and angiogenesis and is associated with relapse through chemoresistance^59–61^. The plasma levels of three ofCSPGs, namely ofCS-CD44, ofCS-SDC1, and ofCS-CSPG4 in six malignant cancer patients, were significantly higher than those of healthy individuals (*P* < 0.001). The ROC curve analysis indicates AUC of all markers are above 0.80 for cancer diagnosis. The ofCS-CD44 showed the highest AUC in the biomarker discovery set (AUC = 0.84) and validation cohort 1 (AUC = 0.83), which achieved a better performance than a recent single biomarker study in colorectal cancer (AUC of 0.62)^62^ and in endometrial cancer (AUC of 0.799)^63^. In an independent larger validation study with 2081 malignant patients and 11,654 healthy controls, we found that the plasma ofCS-CD44 performed well to distinguish various solid malignant tumors with an exception for two types of cancer. In this study, ofCS-CD44 discriminates relatively poor for non-Hodgkin lymphoma and liver hepatocellular carcinoma between cancer patients and healthy controls. The poor performance of other pan-cancer biomarkers in detecting certain cancer was also reported in recent studies^5,9,10,64^. In the DETECT-A study, CancerSEEK was inadequate for hepatobiliary, stomach, neuroendocrine, and sarcoma detection^5^.

It is worth noting that ofCS-CD44 in our study also showed promising early detection value. We found that the plasma ofCS-CD44 levels were increased in stage I and stage II malignant patients, resulting in a similar AUC with all-stage cancer detection. The high plasma ofCS-CD44 is able to successfully predict the increased probability of the presence of malignant cancer in individuals. However, additional multi-center studies are needed to confirm these findings. The ELISA platform of this study offers additional advantages to improve the performance and cost-effectiveness further. For example, the separated assay for different plasma components such as the exosomes specific ofCS might increase the specificity in identifying early cancer or even classifying tumors of unknown primary origin^12,65^. In addition, the high-throughput platform is available or easy to adapt for the current ELISA assay, which will offer robust cost-effectiveness for this pan-cancer biomarker in its clinical utility or population survey. Despite the care in developing and validating the ofCS targeting approach, the study has several limitations. First, the heterogeneity of mysterious oncofetal chondroitin sulfate, including the length of repeating disaccharides chain and the degree of sulfated modification in cancer cells from patients, is not well known. We, therefore, are not able to develop the standard materials that could be used to produce a calibration curve. We set a serial dilution of several high-response plasma samples to each experimental batch, by which we could assess reproducibility among the different batches of test samples. Second, further improvement of the recombinant VAR2CSA quality, particularly to avoid protein aggregation, might increase the binding sensitivity and specificity of the current assay. Third, combination detections of ofCS, ofCSPG, and other promising pan-cancer biomarkers could be done to further improve the performance of the current approach, particularly for cancer types with lower detection sensitivity, such as hepatocellular carcinoma. Finally, the clinical utility of plasma ofCS-CD44 for noninvasive posttreatment surveillance and treatment monitoring in malignant cancer warrants further evaluation in longitudinal studies. Nevertheless, the concurrent targeting of ofCS and its modified glycoproteins enabled a promising biomarker for pan-cancer detection, which could potentially be a cost-effective alternative to the current diagnostic tools.

## Methods

### Expression and purification of rVAR2 recombinant fragments

The codon-optimized VAR2CSA sequences of *P. falciparum* FCR3 and 3D7 (GenBank: ADG23053.1, AFN44727.1) were used to express seven rVAR2 with different CSA binding domains in this study. The rVAR2-1 and rVAR2-2, with hexahistidine tail, were expressed in Baculovirus-infected Sf9 cells (GenScript), as per the previous report^66^. Five other rVAR2 (rVAR2-3 to 7) were expressed in Shuffle T7 E. *coli* (EC2030S, WEIDI). Briefly, the codon-optimized target sequences were cloned into the pGEX-4T1 vector with a modified V5 tag and a 10 × His tag on the C terminus, allowing the expressed proteins to be detected by flow cytometry and immunofluorescence. Bacterial cultures harboring the plasmid were grown overnight and then inoculated into the fresh Terrific Broth (TB) medium. Freshly inoculated cultures were allowed to reach an optical density at 600 nm of 0.6. The cultures were subsequently induced with 0.1 mM isopropyl-β-d-thiogalactoside and grown overnight at 16 °C, followed by pelleting and lysing the cells via a cell disruptor (NJBIO) in lysis buffer (10 mM Na2HPO4, 1.8 mM KH_2_PO_4_, pH 7.1, 140 mM NaCl, 2.7 mM KCl, 5% Glycerol, 2 mM DTT, 1 μM DNase I, 1 mM phenylmethanesulfonylfluoride (PMSF), and protease inhibitor cocktail I, (C0001, TargetMol). The lysate was centrifugated at 40,000 × *g* for 1 h. The supernatant was filtered and incubated in Glutathione resins (GE Healthcare) for 1 h at 22 °C and then washed with wash buffer containing 10 mM Na_2_HPO_4_, 1.8 mM KH_2_PO_4_, pH 7.1, 140 mM NaCl, 2.7 mM KCl, 5% Glycerol, 2 mM DTT until the absorbance at 260 nm spectrophotometer reading reached a value of zero. The recombinant proteins were then digested with glutathione S-transferase (GST)-fused PSP overnight at 4 °C to remove the GST-tag and eluted with elution buffer 1(10 mM Na_2_HPO_4_, 1.8 mM KH_2_PO_4_, pH 7.1, 140 mM NaCl, 2.7 mM KCl, 5% Glycerol), and followed by adjusted the imidazole concentration of eluent to 15 mM and applied to the Ni-NTA column (GE Healthcare) equilibrated with binding buffer containing 20 mM sodium phosphate, pH 7.1, 150 mM NaCl, 15 mM imidazole, and 2 mM β-mercaptoethanol (β-ME). After washing with increasing concentrations of imidazole, up to 150 mM, the protein was finally eluted with 500 mM imidazole and desalted with desalt column (GE Healthcare).

### Surface plasmon resonance (SPR)

SPR was performed on a BIAcore T200 (GE Healthcare, Uppsala, Sweden) as suggested by the manufacturer. Buffers were filtered (0.22 μM) and degassed. The biotin-labeled CSA was performed using an EZ-Link® Hydrazide Biotins (21340, Thermo Fisher). Briefly, the Chondroitin sulfate A (Sigma, cat# c9819, mean molecular mass, 20 kDa) was dissolved in MES Buffer and mixed with the hydrazide-biotin solution and the EDC (1-Ethyl-3-[3-Dimethylaminopropyl]carbodiimide Hydrochloride) solution, and incubated for two hours at room temperature. The biotinylated CSA was separated by a desalting column for biosensor analysis. The biotinylated CSA were immobilized to flow cells in a streptavidin chip. A flow cell was treated with 0.1 μg ml^−1^ biotinylated CSA to reach a ～20 resonance unit(RU) and ~8 RU increase in the sensor chip, which is suitable for smaller and larger molecules according to the formula of theoretical Rmax $({{{{{\rm{R}}}}}}{{{{{\rm{max }}}}}}=\frac{{{{{{\rm{analyte}}}}}\; {{{{\rm{MW}}}}}}}{{{{{{\rm{ligand}}}}}\; {{{{\rm{MW}}}}}}}\times {{{{{\rm{Rl}}}}}}\times {{{{{\rm{Sm}}}}}})$. The protein samples were diluted in HBS-EP buffer (0.01 M HEPES at pH 7.4, 0.15 M NaCl, 3 mM EDTA, and 0.05% surfactant P20) (GE Healthcare, Uppsala, Sweden). Different dilutions of protein samples in the buffer were injected at a flow rate of 30 μL min^−1^. At the end of the sample injection (120 s), the same running buffer was passed over the sensor surface to facilitate dissociation for 600 to 1200 s. After dissociation, the sensor surface was regenerated by injecting 10 mM Glycine-HCl (pH 1.5) to remove the binding proteins. The response was monitored as a function of time (sensor gram) at 25 °C. Multi-concentration data were globally fitted, and residuals were calculated and used to assess the goodness of the fit.

### Cell lines and culture conditions

A panel of cancer cell lines was used in this study. A549, SW480, SW620, HT29, Caco2, LOVO, and HCT116 were cultured in Dulbecco’s Modified Eagle Medium (DMEM, Gibco) with 10% fetal bovine serum (Trace Scientific, Melbourne, Australia). ESCC cell lines (KYSE30 and KYSE180) were generously provided by Professor Guan from SYSUCC and cultured in Roswell Park Memorial Institute 1640 medium (RPMI1640, Gibco) supplemented with 10% fetal bovine serum. All cells were cultured in 5% carbon dioxide at 37 °C in a humidified chamber.

### Immunofluorescence staining

Cells were grown to 70–80% confluency. The attached tumor cells were treated with 4% paraformaldehyde and washed with PBS three times. The slides were blocked with 10% goat serum for 1 h at room temperature and incubated with rVAR2 in PBS containing 2% Bull Serum Albumin (BSA, MP) for 1 h at room temperature. Following staining with FITC conjugated anti-V5 antibody (1:500, R963-25, Thermo Fisher) and DAPI (G1012, Servicebio), images were taken by laser scanning confocal microscope (LSM 880, ZEISS).

Colocalization was further determined using a modified protocol as previously reported^23^. Briefly, the slides with fixed cancer cells were incubated with primary antibodies of anti-CD44 (1:200, Proteintech, Cat#60224-1-Ig), -CSPG4 (1:200, Proteintech, Cat#55027-1-AP), and -SDC1 (1:200, Proteintech, Cat#67155-1-Ig), respectively, at 4 °C overnight in a dark humidified chamber. The cells were washed thrice in PBS and incubated with anti-rabbit IgG (H + L), F(ab’)2 Fragment (Cell Signaling, Cat#4413 or 4409) in blocking buffer (1:1000 dilution) for 1 h at room temperature in the dark. After CSPGs capture, the coverslip was washed thrice with PBS and incubated with rVAR2-3 (3 μg mL^−1^) and V5 Tag Antibody (1:500, Invitrogen, Cat#R963-25) for ofCS detection. Similarly, the coverslip was washed thrice with PBS and incubated with DAPI (Servicebio, Cat#G1012) for DNA staining. The coverslips were washed and then mounted to the cover slide with an antifade mounting medium (Absin, Cat# abs9234) and left to cure for 24 h. Images were taken on a Zeiss LSM 880 confocal microscope at × 40 magnification. Colocalization was measured using the colocalization module in Zen Blue (v2.5, Zen blue edition, Zeiss, Germany) software. An entire field of view is analyzed on a pixel-by-pixel basis. A modified Pearson’s correlation coefficient (R) is generated to determine the colocalization of two channels. R is reported for an entire image of 1024 px by 1024 px. *R* = 1 represents perfect correlation.

### Flow cytometry

The culture cells were dissociated by Gentle Cell Dissociation Reagent (07174, Stem cell) at room temperature and washed with PBS by centrifugation at 450 × *g* for 5 min. After blocking with 10% goat serum for 1 h at room temperature using end-over-end rotation, the cells were incubated with rVAR2 (200 to 25 nM, a serial of twofold dilution) at room temperature for 1 h, and 5% BSA was used as the negative control. The cells were incubated with FITC conjugated anti-V5 antibody (1:500, R963-25, Thermo Fisher) and suspended in 5% BSA before the binding of rVAR2 was detected using CytoFLEX (Beckman 2.2). An exemplification of the gating strategy for flow cytometry was shown in a source data file.

### Patients and sample collection

Malignant tumor patients were recruited randomly and retrospectively from different cancer centers in China: the Sun Yat-Sen University Cancer Center (SYSUCC) (for biomarker discovery and validation) and Tianjin Cancer Center (for external validation). In this study, the inclusion criteria for malignant tumor patients’ specimens were as follows: (1) all subjects were histologically confirmed with a malignant tumor, (2) the malignant tumor patients did not receive any clinical treatment such as chemotherapy or radiotherapy before blood collection or surgery. Clinical and demographic characteristics, such as age, gender, American Joint Committee on Cancer (AJCC) stage for a solid malignant tumor, and Ann Arbor Staging System for non-Hodgkin lymphoma were obtained from medical records. From Sun Yat-Sen University Cancer Center, a total of 165 patients who were diagnosed in 2019 were recruited as cases in the biomarker discovery set, which consist of tongue cancer (*n* = 29), NPC (*n* = 27), ESCC (*n* = 27), ccRCC (*n* = 27), CRC (*n* = 26), and BLCA (*n* = 29), respectively. A total of 2081 malignant tumor patients who were diagnosed between Jan. 2013 to Dec. 2015 were used for the biomarker validation cohort 2, which include 11 different cancers: BLCA (*n* = 86), ESCC (*n* = 257), LC (*n* = 274), GC (*n* = 264), NPC (*n* = 223), PC (*n* = 75), CRC (*n* = 200), BC (*n* = 91), CC (*n* = 177), NHL (*n* = 209), and HCC (*n* = 225). In the validation cohort 1 study, 400 cancer samples diagnosed from 2015–2022 were collected from Tianjin Cancer Center and 21 regions in China other than Guangzhou to validate the performance of all three ofCS-CSPGs for pan-cancer detection. The eight cancers include bladder cancer (*n* = 50), esophageal squamous cell carcinoma (*n* = 50), gastric cancer (*n* = 50), lung cancer (*n* = 50), colorectal carcinoma (*n* = 50), pancreatic cancer (*n* = 50), breast cancer (*n* = 50), and cervical cancer (*n* = 50), respectively.

Healthy subjects without evidence of any cancer were selected from Guangdong Biobank Cohort Study (ChiCTR1800015736) and the routine general health check-ups in both cancer centers. We randomly selected 302 healthy participants as controls in the biomarker discovery set, 11,654 healthy participants in the validation cohort, and 200 healthy participants in the external validation cohort (Demography characteristics and clinical information of individuals were summarized in Supplementary Tables 1, 2). This study was approved by the Ethics committees of SYSUCC (approval number: GZJZ-SB2016-022) and Tianjin cancer center (approval number: E20210664), and written informed consent was obtained from all participants to use their plasma samples. Two blood samples from Acute Lymphocytic Leukemia patients and a healthy donor were collected for FACS analysis.

All blood samples were collected according to the standard operating procedure. Five to ten mL venous blood sample was collected in EDTA-containing tubes and immediately centrifuged at 4000 × *g* for 10 min for plasma collection. Plasma aliquots were stored at −80 °C immediately or within 4 h after blood collection.

### Enzyme-linked immunosorbent assay

Anti-CD44, Anti-SDC1, and Anti-CSPG4 antibodies were purchased from Proteintech (Proteintech, USA). The sandwich ELISA was developed by using Anti-CD44 (3 ug/ml), Anti-SDC1 (3 ug/ml), Anti-CSPG4 (3 ug/ml) antibodies, rVAR2-5 (3 ug/ml), as the coating proteins, and HRP labeling rVAR2-3, as the detecting proteins to quantify the ofCS or ofCS modified proteoglycans in the plasma. The antibodies were diluted in a coating buffer containing 50 mmol L^−1^ carbonate buffer, pH 9.6 (C3041, Sigma), and 96-well microplates were coated with 50 μL per well of the diluted antibodies at 4 °C overnight. The coated 96-well plates were washed three times with wash buffer containing 0.05%Tween-20 in TBS (TBST), followed by 200 μL blocking buffer (1% gelatin in TBST) per well for 2-h incubation at 37 °C. After three washes with the washing buffer, 50 μL serum (1:25 dilution) was added and incubated at room temperature. The HRP-labeled recombinant rVAR2-3 with a final concentration of 0.1 ng μL^−1^ was added into the wells after five washes with the wash buffer. Followed by 1 h of incubation at room temperature and five washes with the wash buffer, the plates were supplemented with 100 μL Tetramethylbenzidine solution (TMB, KGP125100, KeyGEN). Finally, the reaction was terminated by adding stop solution (KGP12710, KeyGEN), and optical density measurements were taken at 450 nm on a microplate Spectrophotometer (Epoch™ 2, BioTek). Plasma samples from different malignancies and healthy controls were randomized and tested blindly throughout the whole process.

For sensitivity assay, anti-CD44 (3 ug/ml), -CSPG4 (3 ug/ml), and -SDC1(3 ug/ml) antibodies were coated and incubated at 4 °C overnight. The 96-well plate was washed and blocked with TBST containing 1% gelatin. The cancer cell spiked samples were prepared using SW480 cell lines ranging from 0 to 21,500 cells μL^−1^. After three washes, a 50 μL diluted sample was added and incubated at room temperature. For specificity assay, decorin (Sigma, D8428), chondroitinase-treated decorin, CSA, or BSA were directly coated on the ELISA plate as previously reported^37^. The plasma samples and cell lysates of SW480 were pretreated with 1 Unit mL^−1^ chondroitinase ABC at 37 °C for 2 h. Digested CS chains were ultrafiltered by an ultrafiltration tube (Millipore, USA), and the remaining proteins were added to an anti-CD44-coated 96-well plate. In the competitive assay, gradient dilution CSA (3.75 × 10^−5^–7.5 mg mL^−1^) was added to an anti-CD44-coated 96-well plate for competitive incubation. The total level of CD44 was measured using a commercial kit (ab45912, Abcam).

### Statistics and reproducibility

We run the power analysis with sample size calculation in the study design. We assumed the biomarker with ORs of 2, 3, and 5, and the case-control ratio of 1:10, 1:3, 1:1, and 2:1. We used the R package “sample size logistic case-control” to perform power and sample size analysis at the setting of the type one error of 0.05 and the disease prevalence of 100/100000. With the most conservative estimation (OR = 2 and case-control ratio = 1:10), the total sample size required is found to be about 250 to achieve a power of 0.9. In the discovery stage, we selected a larger sample size of about 30 for each cancer type and 302 for the healthy control to ensure sufficient power. To validate the stability and consistent discrimination ability of the identified biomarker, we enlarged our sample size to a total of 400 cases and 200 controls (validation cohort 1) and 2081 cases and 11,654 controls (validation cohort 2). Graphing and statistical analyses were performed using R software (R-3.6.0) and GraphPad Prism (Version 8.4.3). The patient cohort was described using frequencies for categorical variables. The area under the curve (AUC) in ROC analysis was used to evaluate the predictive performance of biomarkers to distinguish malignant patients from healthy controls. The logistic regression model was developed and calculated as follows: logit (score) = α + β1 × ofCS modified protein + β2 × gender + β3 × age, where α represented the intercept, β represented the regression coefficients of each variate. We also used logistic regression models to assess the associations between plasma ofCS-CD44 levels and the malignant tumor and to estimate odds ratios (ORs) and 95% CIs adjusted for age and sex. The plasma ofCS-CD44 levels were categorized into ten groups according to the decile of the healthy control group, and the lowest 20 percent sample was treated as the reference group for pan-cancer analysis. Additionally, we set the healthy female person as the control group for breast cancer and cervical cancer. Using logistic regression analysis, we compared the risk of participants in each decile with the reference group, respectively. The ELISA linear range of VAR2CSA binding to ofCS-CD44, -CSPG4, and –SDC1 was analyzed by the 4PL sigmoidal nonlinear regression model. Nonparametric *t*-tests were used to compare the control and cancer cell spiked samples. The SDS-PAGE and Western blotting have been performed three times independently with similar results.

### Reporting summary

Further information on research design is available in the Nature Portfolio Reporting Summary linked to this article.

## Supplementary information

Supplementary Information

Reporting Summary

## Data Availability

The ELISA data generated in this study have been deposited in the Figshare database at 10.6084/m9.figshare.21723716 [https://figshare.com]. The baseline patient information is provided in the Supplementary Information. The summary statistics that support the findings of this study are provided in the Supplementary Information and Source Data file. Source data are provided with this paper.

## Source data

Source Data

## Code availability

The code supporting the conclusions of this study is available from the corresponding author upon reasonable request.
