# Supplementary material for: Establishment and validation of a plasma oncofetal chondroitin sulfated proteoglycan for pan-cancer detection
Source: Nat Commun. 2023 Feb 6;14:645. doi: 10.1038/s41467-023-36374-7 (PMC9902466; doi:10.1038/s41467-023-36374-7)
Supplement: Supplementary file 1 — Supplementary Information [file 41467_2023_36374_MOESM1_ESM.pdf]

Establishment and validation of a plasma oncofetal chondroitin sulfated proteoglycan for pan-cancer detection

Supplementary Figure.1 Validation and binding affinity identification of recombinant VAR2CSA.

Supplementary Figure.2 Binding specificity of VAR2CSA with ofCSPGs and decorin.

Supplementary Figure.3 Diagnosis performance of ofCS/ofCSPGs and detection of total CD44 level.

Supplementary Figure.4 Plasma ofCS-CD44 levels (median with inter-quartile range) in different cancer stages.

Supplementary Table 1 Characteristics of the individuals enrolled for the biomarker discovery set

Supplementary Table 2 Characteristics of the individuals enrolled for the biomarker validation set

Supplementary Table 3 The diagnosis performance using combination of plasma ofCS/ofCSPGs at discovery set

Supplementary Table 4 Odds ratios (ORs) of increased ofCS-CD44 with malignant tumor

Supplementary Table 5 Sensitivity and specificity of ofCS-CD44 in different cancer stages

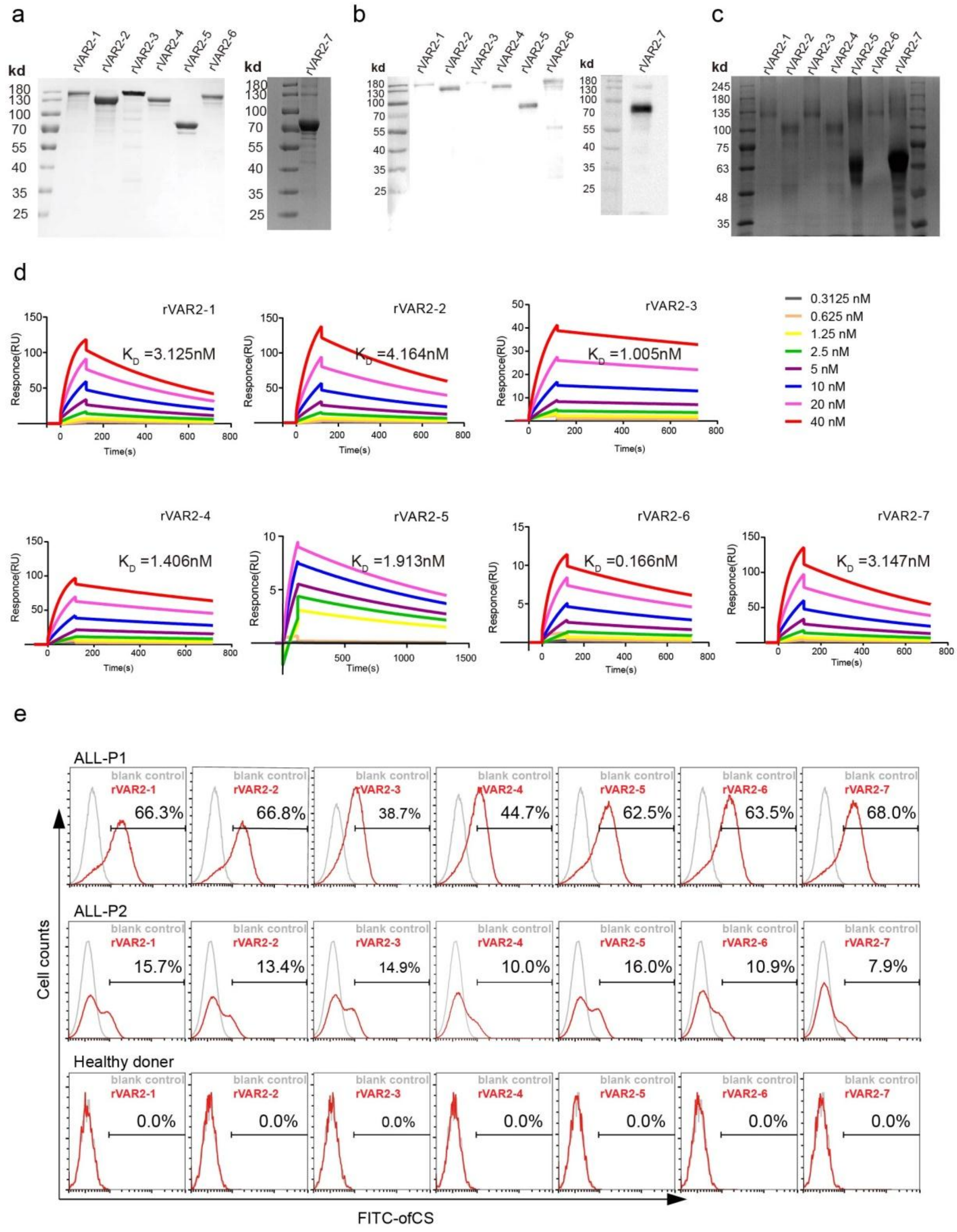

Supplementary Fig.1 Validation and binding affinity identification of recombinant VAR2CSA. (a) SDS-PAGE and Coomassie brilliant blue staining of rVAR2- (#1-7). (b) Western-Blot of rVAR2 (#1-7) incubated with anti-V5 antibody and detected by HRP

labeled anti-mouse IgG antibody. (c) non-reducing SDS-PAGE and Coomassie brilliant blue staining of rVAR2 (#1-7). (d) Sensorgram showing binding between rVAR2 (#1-7) and CSA measured in Response (RU) as a function of time (in seconds) using the indicated concentrations of recombinant protein. K_D values were presented. Due to the presence of aggregated rVar2, a portion of the kD might be calculated on subfraction of the added protein. (e) FACS flow of white blood cells from two acute lymphoblastic leukemia patients as well as one healthy donor detected with 200nM rVAR2. ALL, acute lymphoblastic leukemia. P1, Patient 1. P2, Patient 2.

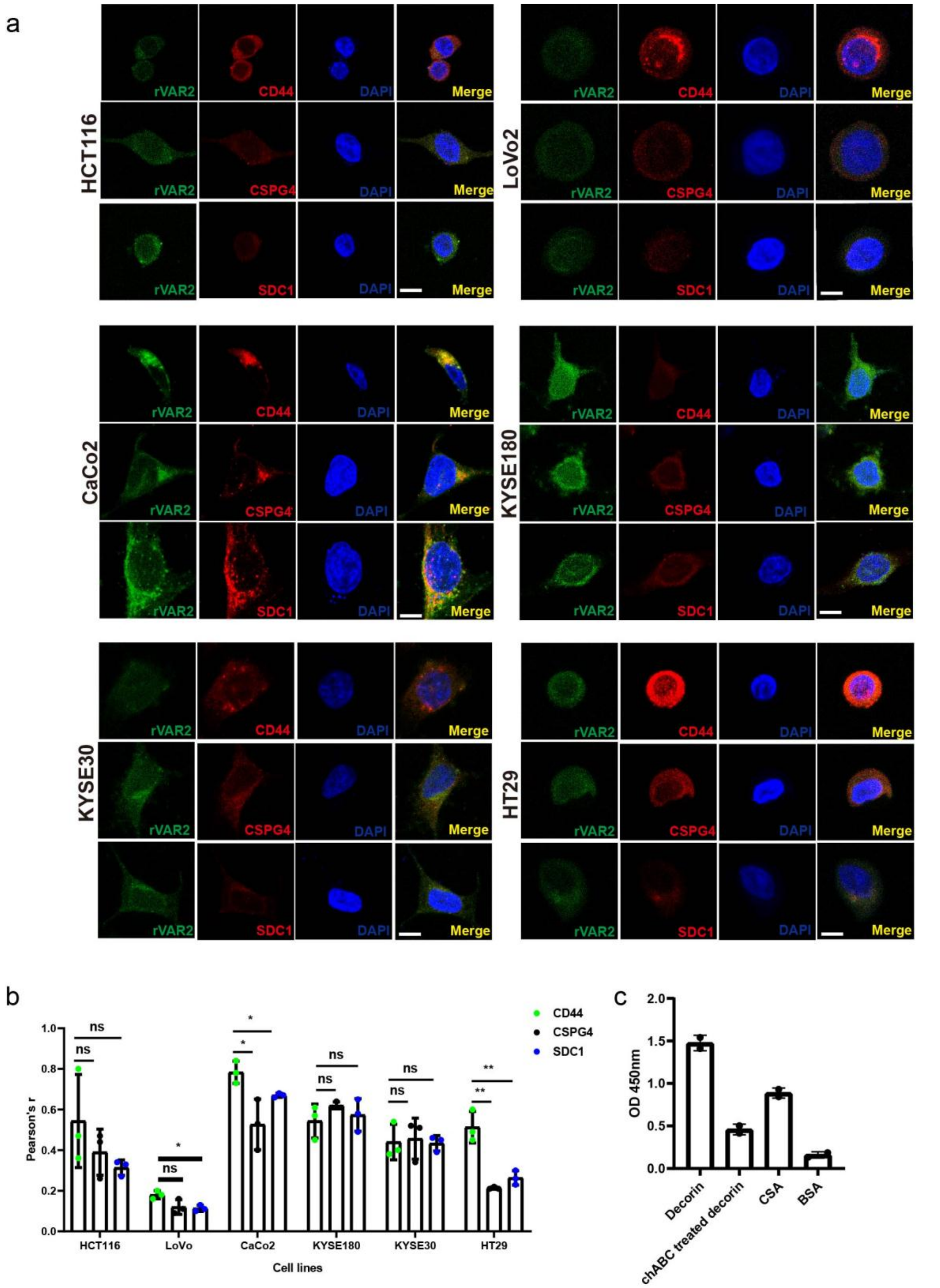

Supplementary Figure.2 Binding specificity of VAR2CSA with ofCSPGs and decorin. (a) Colocalization of ofCS with core proteins detected by rVar2-3 and anti-CD44, CSPG4, and SDC1 antibodies in HCT116, LoVo2, CaCo2, KYSE180,

KYSE30, and HT29 cell lines. Scale bar= 10μm. (b) Colocalization analysis of ofCS glycan with CSPGs (CD44, CSPG4, and SDC1) (n=3 biologically independent experiments). Pearson's correlation coefficient was calculated using Zen (Black edition) software. *P*=0.3575 and *P*=0.1612 for CD44 vs. CSPG4 and CD44 vs. SDC1 in HCT116, *P*=0.0653 and *P*=0.0101 for CD44 vs. CSPG4 and CD44 vs. SDC1 in LoVo, *P*=0.0313 and *P*=0.0247 for CD44 vs. CSPG4 and CD44 vs. SDC1 in CaCo2, *P*=0.1935 and *P*=0.6764 for CD44 vs. CSPG4 and CD44 vs. SDC1 in KYSE180, *P*=0.8394 and *P*=0.9092 for CD44 vs. CSPG4 and CD44 vs. SDC1 in KYSE30, *P*=0.0026 and *P*=0.0071 for CD44 vs. CSPG4 and CD44 vs. SDC1 in HT29, respectively. The t-test was used, all the data were two-sided. Data are shown as mean ± SD, ns. no significant, *. p value < 0.05, **. p value <0.01. (c) Var2CSA recombinants bind specifically to decorin (n=2 biologically independent experiments, data are shown as mean ± SD).

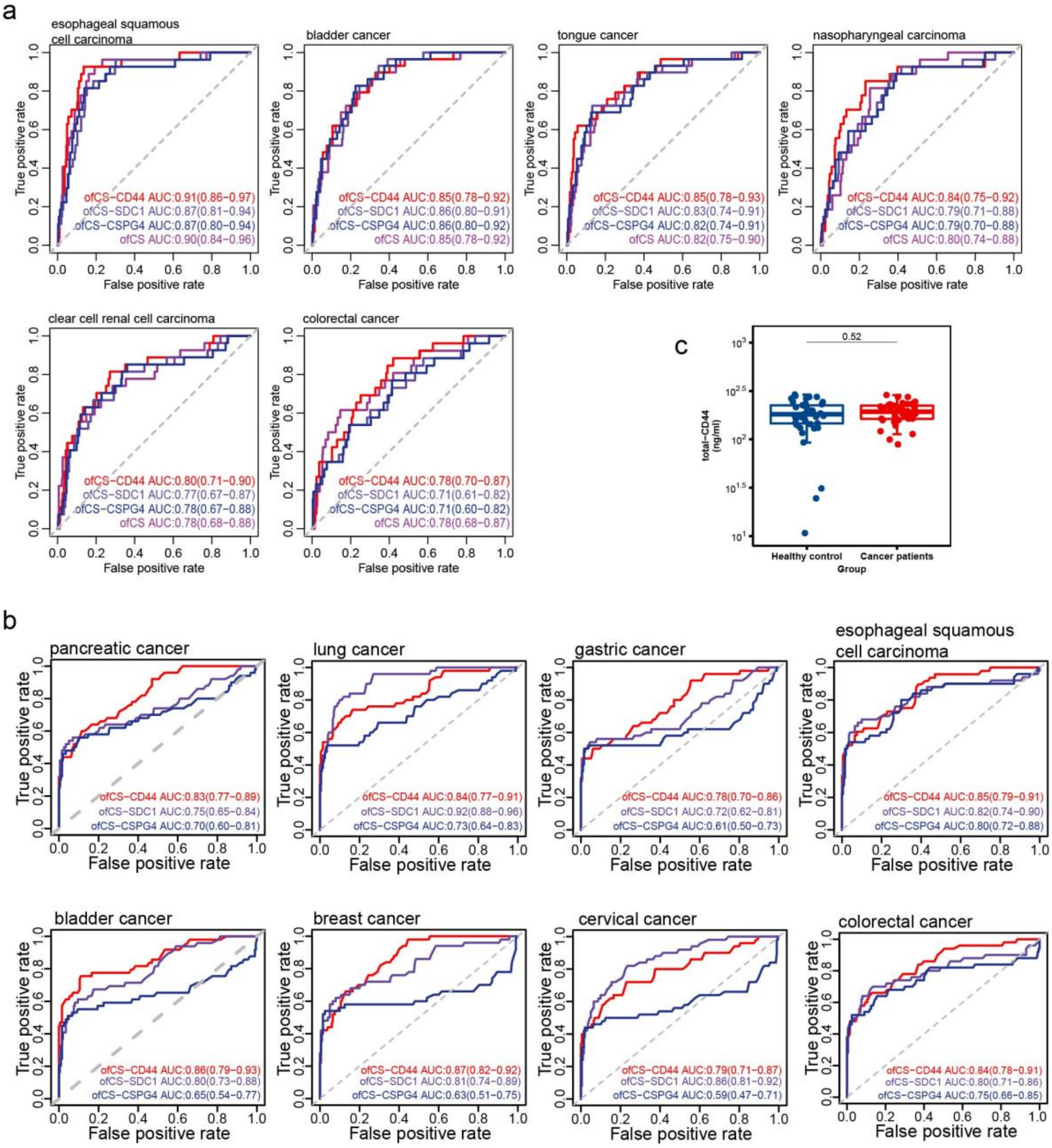

Supplementary Figure.3 Diagnosis performance of ofCS/ofCSPGs and detection of total CD44 level. (a) ROC analysis of the diagnosis performance of ofCS-CD44, SDC1, CSPG4, and total ofCS for different cancer types in the discovery set consisting 6 cancer types. (b) ROC analysis of the diagnosis performance of ofCS-CD44, SDC1 and CSPG4 for different cancer types in the validation stage 1 consisting 8 cancer types. (c) The total level of CD44 in healthy controls (n=41) and cancer patients (n=41). The middle line in the boxplot displays the median, the box indicates the first and third quartile. The Wilcox test was used and the test was two-sided.

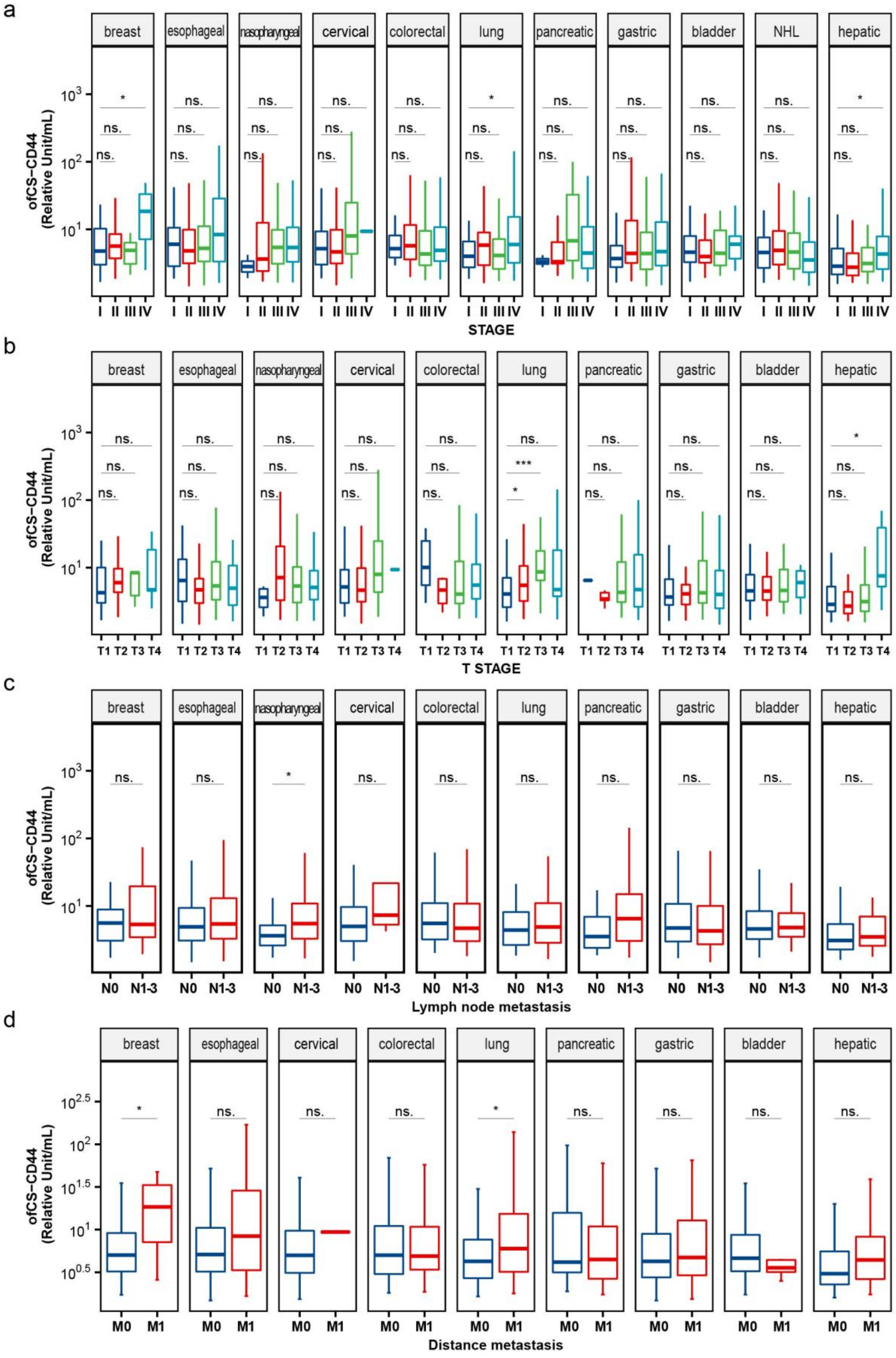

a
breast
esophageal
nasopharyngeal
cervical
colorectal
lung
pancreatic
gastric
bladder
NHL
hepatic
ofCS−CD44
(Relative Unit/mL)
I II III IV
STAGE
b
breast
esophageal
nasopharyngeal
cervical
colorectal
lung
pancreatic
gastric
bladder
hepatic
ofCS−CD44
(Relative Unit/mL)
T1 T2 T3 T4
T STAGE
c
breast
esophageal
nasopharyngeal
cervical
colorectal
lung
pancreatic
gastric
bladder
hepatic
ofCS−CD44
(Relative Unit/mL)
N0 N1-3
Lymph node metastasis
d
breast
esophageal
cervical
colorectal
lung
pancreatic
gastric
bladder
hepatic
ofCS−CD44
(Relative Unit/mL)
M0 M1
Distance metastasis

Supplementary Figure.4 Plasma ofCS-CD44 levels (median with inter-quartile range) in different cancer stages. (a) 11 cancer types (n=2081). (b) T stages of 10 cancer types except for NHL (n=1872). (c) Lymph node metastasis status of 10 cancer types except for NHL (n=1872). (d) Distance metastasis status of 9 cancer types except for NHL and nasopharyngeal carcinoma (n=1649). Value in each figure was compared with the first group. The middle line in the boxplot displays the median, the box indicates the first and third quartile. The Wilcox test was used. All the tests were two-sided. NHL. Non-Hodgkin lymphoma, ns. no significant, *. p value <0.05, ***. p value <0.001.

Supplementary Table 1 Characteristics of the individuals enrolled for the biomarker discovery set

	healthy control	tongue cancer	nasopharyngeal carcinoma	esophageal squamous cell carcinoma	clear cell renal cell carcinoma	colorectal cancer	bladder cancer
Samples, *N*	302	29	27	27	27	26	29
Average age, y (SD)	51.6 (19.1)	50.1 (13.1)	46.7 (8.7)	61.7 (7.3)	52.4 (10.0)	61.3 (9.4)	60.4 (11.7)
Sex, N (%)							
Female	181 (59.9)	6 (20.7)	7 (25.9)	4 (14.8)	8 (29.6)	10 (38.5)	4 (13.8)
Male	121 (40.1)	23 (79.3)	20 (74.1)	23 (85.2)	19 (70.4)	16 (61.5)	25 (86.2)
T stage, N (%)							
Tis	/	/	/	/	/	/	11 (37.9)
1	/	15 (51.7)	/	2 (7.4)	21 (77.8)	2 (7.7)	8 (27.6)
2	/	8 (27.6)	4 (14.8)	3 (11.1)	2 (7.4)	3 (11.5)	8 (27.6)
3	/	4 (13.8)	19 (70.4)	19 (70.4)	4 (14.8)	18 (69.2)	1 (3.45)
4	/	2 (6.90)	4 (14.8)	3 (11.1)	/	3 (11.5)	1 (3.45)
x	/	/	/	/	/	/	/
N stage, N (%)							
0	/	20 (69.0)	/	7 (25.9)	27 (100.0)	14 (53.8)	27 (93.1)
1	/	5 (17.2)	10 (37.0)	8 (29.6)	/	11 (42.3)	1 (3.45)
2	/	4 (13.8)	12 (44.4)	7 (25.9)	/	1 (3.9)	1 (3.45)
3	/	/	5 (18.5)	5 (18.5)	/	/	/
x	/						
M stage, N (%)							
0	/	29 (100.0)	25 (92.6)	26 (96.3)	25 (92.6)	26 (100.0)	29 (100.0)
1	/	/	2 (7.4)	1 (3.7)	2 (7.4)	/	/
Cancer stages, N (%)							

Supplementary Table 1 Characteristics of the individuals enrolled for the biomarker discovery set

	healthy control	tongue cancer	nasopharyngeal carcinoma	esophageal squamous cell carcinoma	clear cell renal cell carcinoma	colorectal cancer	bladder cancer
Tis	/	/	/	/	/	/	11 (37.9)
Stage I	/	13 (44.8)	/	2 (7.4)	21 (77.8)	4 (15.4)	8 (27.6)
Stage II	/	7 (24.1)	1 (3.7)	6 (22.2)	1 (3.7)	10 (38.5)	8 (27.6)
Stage III	/	5 (17.2)	16 (59.3)	18 (66.7)	3 (11.1)	12 (46.2)	/
Stage IV	/	4 (13.8)	10 (37.0)	1 (3.7)	2 (7.4)	/	2 (6.90)

Supplementary Table 2 Characteristics of the individuals enrolled for the biomarker validation set

	healthy control	bladder cancer	esophageal squamous cell carcinoma	gastric cancer	nasophary-ngeal carcinoma	lung cancer	colorectal cancer	pancreatic cancer	breast cancer	cervical cancer	non-Hodgkin's lymphoma	hepatocellular carcinoma
**validation cohort-1**												
Samples, *N*	200	50	50	50	/	50	50	50	50	50	/	/
Average age, y (SD)	51.6(13.9)	64.6(14.0)	55.8(12.3)	61.9(12.4)	/	61.6(14.6)	60.3(14.3)	58.3(13.2)	54.2(12.1)	49.3(14.5)	/	/
Sex, N (%)												
Female	101(50.5)	15(30.0)	8(16.0)	22(44.0)	/	14(28.0)	21(42.0)	23(46.0)	50(100.0)	50(100.0)	/	/
Male	99(49.5)	35(70.0)	42(84.0)	28(56.0)	/	36(72.0)	29(58.0)	27(54.0)	/	/	/	/
**validation cohort-2**												
Samples, *N*	11654	86	257	264	223	274	200	75	91	177	209	225
Average age, y (SD)	52.9 (15.0)	62.8 (10.7)	60.7 (8.5)	56.1 (10.9)	46.5 (8.7)	56.8 (10.2)	55.2 (10.4)	55.5 (11.1)	47.6 (9.6)	48.9 (8.8)	51.6 (12.0)	53.3 (10.4)
Sex, N (%)												
Female	8254 (70.8)	18 (20.9)	84 (32.7)	78 (29.5)	58 (26.0)	88 (32.1)	91 (45.5)	31 (41.3)	89 (97.8)	177 (100.0)	84 (40.2)	48 (21.3)
Male	3400 (29.2)	68 (79.1)	173 (67.3)	186 (70.5)	165 (74.0)	186 (67.9)	109 (54.5)	44 (58.7)	2 (2.2)	/	125 (59.8)	177 (78.7)
T stage, N (%)												
Tis	/											
1	/	45 (52.3)	28 (10.9)	42 (15.9)	6 (2.7)	154 (56.2)	6 (3.0)	1 (1.3)	36 (39.6)	104 (58.8)	/	72 (32.0)
2	/	20 (23.3)	47 (18.3)	25 (9.5)	27 (12.1)	83 (30.3)	16 (8.0)	11 (14.7)	45 (49.5)	68 (38.4)	/	41 (18.2)
3	/	13 (15.1)	142 (55.3)	76 (28.8)	136 (61.0)	20 (7.3)	95 (47.5)	22 (29.3)	5 (5.5)	4 (2.3)	/	97 (43.1)
4	/	7 (8.1)	30 (11.7)	89 (33.7)	54 (24.2)	11 (4.0)	75 (37.5)	31 (41.3)	5 (5.5)	1 (0.6)	/	5 (2.2)
x	/	1 (1.2)	10 (3.9)	32 (12.1)	/	6 (2.2)	8 (4.0)	10 (13.3)	/	/	/	10 (4.4)

Supplementary Table 2 Characteristics of the individuals enrolled for the biomarker validation set

	healthy control	bladder cancer	esophageal squamous cell carcinoma	gastric cancer	nasophary-ngeal carcinoma	lung cancer	colorectal cancer	pancreatic cancer	breast cancer	cervical cancer	non-Hodgkin's lymphoma	hepatocellular carcinoma
N stage, N (%)												
0	/	76 (88.4)	93 (36.2)	76 (28.8)	13 (5.8)	89 (32.5)	59 (29.5)	22 (29.3)	51 (56.0)	173 (97.7)	/	/
1	/	4 (4.6)	89 (34.6)	36 (13.6)	88 (39.5)	73 (26.6)	78 (39.0)	40 (53.3)	22 (24.2)	1 (0.6)	/	194 (86.2)
2	/	2 (2.3)	37 (14.4)	48 (18.2)	92 (41.2)	93 (33.9)	54 (27.0)	2 (2.7)	13 (14.3)	/	/	29 (12.9)
3	/	3 (3.5)	24 (9.3)	80 (30.3)	30 (13.5)	18 (6.6)	1 (0.5)	1 (1.3)	5 (5.49)	/	/	2 (0.9)
x	/	1 (1.2)	14 (5.5)	24 (9.1)	/	1 (0.4)	8 (4.0)	10 (13.3)	/	3 (1.7)	/	
M stage, N (%)												
0	/	82 (95.3)	225 (87.5)	192 (72.7)	223 (100.0)	183 (66.8)	147 (73.5)	32 (42.7)	82 (90.1)	176 (99.4)	/	205 (91.1)
1	/	4 (4.7)	32 (12.5)	72 (27.3)	/	91 (33.2)	53 (26.5)	43 (57.3)	9 (9.9)	1 (0.6)	/	20 (8.9)
Cancer stages, N (%)												
Tis	/	/	/	/	/	/	/	/	/	/	/	/
Stage I	/	44 (51.2)	27 (10.5)	50 (18.9)	2 (0.9)	71 (25.9)	4 (2.0)	3 (4.0)	30 (33.0)	104 (58.8)	30 (14.4)	73 (32.4)
Stage II	/	21 (24.4)	76 (29.6)	48 (18.2)	13 (5.8)	36 (13.1)	43 (21.5)	10 (13.3)	38 (41.8)	68 (38.4)	64 (30.6)	36 (16.0)
Stage III	/	12 (14.0)	122 (47.5)	94 (35.6)	131 (58.7)	76 (27.7)	100 (50.0)	19 (25.3)	14 (15.4)	4 (2.3)	58 (27.8)	76 (33.8)
Stage IV	/	9 (10.5)	32 (12.5)	72 (27.3)	77 (34.5)	91 (33.2)	53 (26.5)	43 (57.3)	9 (9.9)	1 (0.6)	57 (27.3)	40 (17.8)

Supplementary Table 3 The diagnosis performance using combination of plasma ofCS/ofCSPGs at discovery set

ofCSPGs used in logistic regression model	AUC (95%CI) of Model1 [a]	AUC (95%CI) of Model2 [b]
ofCS-CD44 + ofCS	0.81 (0.77-0.85)	0.85 (0.81-0.89)
ofCS-SDC1 + ofCS	0.79 (0.75-0.83)	0.84 (0.80-0.88)
ofCS-CSPG4 + ofCS	0.78 (0.74-0.83)	0.84 (0.80-0.87)
ofCS-CD44 + ofCS-CSPG4	0.81 (0.77-0.85)	0.85 (0.81-0.88)
ofCS-CD44 + ofCS-SDC1	0.81 (0.76-0.85)	0.85 (0.81-0.88)
ofCS-SDC1 + ofCS-CSPG4	0.75 (0.71-0.80)	0.81 (0.77-0.85)
ofCS-CD44 + ofCS-SDC1 + ofCS	0.81 (0.77-0.85)	0.85 (0.82-0.89)
ofCS-CD44 + ofCS-CSPG4 + ofCS	0.81 (0.77-0.85)	0.85 (0.82-0.89)
ofCS-SDC1 + ofCS-CSPG4 + ofCS	0.79 (0.75-0.83)	0.84 (0.80-0.88)
ofCS-CD44 + ofCS-CSPG4 + ofCS-SDC1	0.81 (0.76-0.85)	0.85 (0.81-0.88)
ofCS-CD44 + ofCS-CSPG4 + ofCS-SDC1 + ofCS	0.81 (0.77-0.85)	0.85 (0.82-0.89)

[a] Model 1: logistic regression model established based on the ofCSPGs only

[b] Model 2: logistic regression model established based on the ofCSPGs, age and sex.

Supplementary Table 4 Odds ratios (ORs) of increased ofCS-CD44 with malignant tumor

Cancer type	ofCS-CD44 decile	Healthy person, *N* (%)	case, *N* (%)	OR [a]	95% CI [b]	*P* value [c]
bladder cancer	0-30%	3496 (30.0)	4 (4.7)	1.0	reference	reference
	30-40%	1161 (10.0)	5 (5.8)	3.7	1.1-12.3	3.03E-02
	40-50%	1170 (10.0)	6 (7.0)	3.7	1.1-12.2	3.07E-02
	50-60%	1165 (10.0)	13 (15.1)	8.2	2.9-22.9	5.73E-05
	60-70%	1166 (10.0)	14 (16.3)	9.7	3.5-26.5	1.07E-05
	70-80%	1165 (10.0)	12 (14.0)	7.4	2.6-20.8	1.58E-04
	80-90%	1165 (10.0)	16 (18.6)	9.5	3.4-25.9	1.30E-05
	90-100%	1166 (10.0)	16 (18.6)	9.5	3.5-26.0	1.25E-05
esophageal squamous cell carcinoma	0-30%	3496 (30.0)	12 (4.7)	1.0	reference	reference
	30-40%	1161 (10.0)	18 (7.0)	4.5	2.2-9.3	3.06E-05
	40-50%	1170 (10.0)	21 (8.2)	4.9	2.5-9.9	7.26E-06
	50-60%	1165 (10.0)	23 (9.0)	5.4	2.7-10.7	1.15E-06
	60-70%	1166 (10.0)	33 (12.8)	8.2	4.3-15.6	1.49E-10
	70-80%	1165 (10.0)	41 (16.0)	9.4	5.0-17.6	2.92E-12
	80-90%	1165 (10.0)	38 (14.8)	8.7	4.6-16.5	2.58E-11
	90-100%	1166 (10.0)	71 (27.6)	16.2	8.9-29.5	7.03E-20
gastric cancer	0-30%	3496 (30.0)	17 (6.4)	1.0	reference	reference
	30-40%	1161 (10.0)	24 (9.1)	4.1	2.2-7.6	5.50E-06
	40-50%	1170 (10.0)	27 (10.2)	4.5	2.5-8.2	1.05E-06
	50-60%	1165 (10.0)	36 (13.6)	6.0	3.4-10.6	7.32E-10
	60-70%	1166 (10.0)	30 (11.4)	5.4	3.0-9.6	1.76E-08
	70-80%	1165 (10.0)	31 (11.7)	5.2	2.9-9.3	3.46E-08
	80-90%	1165 (10.0)	35 (13.3)	5.7	3.2-10.1	3.22E-09
	90-100%	1166 (10.0)	64 (24.2)	10.3	6.1-17.5	6.62E-18
nasopharyngeal carcinoma	0-30%	3496 (30.0)	9 (4.0)	1.0	reference	reference
	30-40%	1161 (10.0)	13 (5.8)	4.0	1.8-9.1	8.66E-04
	40-50%	1170 (10.0)	18 (8.1)	5.3	2.4-11.5	3.01E-05
	50-60%	1165 (10.0)	29 (13.0)	8.8	4.3-18.2	3.73E-09
	60-70%	1166 (10.0)	24 (10.8)	7.9	3.8-16.6	3.78E-08
	70-80%	1165 (10.0)	34 (15.2)	10.8	5.3-22.0	5.74E-11
	80-90%	1165 (10.0)	40 (17.9)	11.8	5.8-23.7	5.82E-12
	90-100%	1166 (10.0)	56 (25.1)	16.3	8.2-32.2	1.01E-15
lung cancer	0-30%	3496 (30.0)	15 (5.5)	1.0	reference	reference
	30-40%	1161 (10.0)	27 (9.9)	5.3	2.8-9.8	1.57E-07
	40-50%	1170 (10.0)	25 (9.1)	4.7	2.5-8.8	1.71E-06
	50-60%	1165 (10.0)	33 (12.0)	6.2	3.4-11.3	2.73E-09

Supplementary Table 4 Odds ratios (ORs) of increased ofCS-CD44 with malignant tumor

Cancer type	ofCS-CD44 decile	Healthy person, *N* (%)	case, *N* (%)	OR [a]	95% CI [b]	*P* value [c]
	60-70%	1166 (10.0)	27 (9.9)	5.5	3.0-10.1	6.56E-08
	70-80%	1165 (10.0)	39 (14.2)	7.3	4.0-13.1	3.35E-11
	80-90%	1165 (10.0)	48 (17.5)	8.8	5.0-15.6	9.46E-14
	90-100%	1166 (10.0)	60 (21.9)	10.9	6.2-19.0	5.11E-17
colorectal cancer	0-30%	3496 (30.0)	5 (2.5)	1.0	reference	reference
	30-40%	1161 (10.0)	12 (6.0)	6.5	2.5-17.2	1.56E-04
	40-50%	1170 (10.0)	16 (8.0)	8.0	3.1-20.4	1.53E-05
	50-60%	1165 (10.0)	32 (16.0)	16.1	6.7-38.6	4.40E-10
	60-70%	1166 (10.0)	28 (14.0)	15.1	6.2-36.3	1.53E-09
	70-80%	1165 (10.0)	30 (15.0)	15.3	6.3-36.7	1.14E-09
	80-90%	1165 (10.0)	25 (12.5)	12.3	5.0-30.1	3.72E-08
	90-100%	1166 (10.0)	52 (26.0)	25.5	10.9-59.6	7.20E-14
pancreatic cancer	0-30%	3496 (30.0)	6 (8.0)	1.0	reference	reference
	30-40%	1161 (10.0)	6 (8.0)	3.0	1.0-8.6	4.06E-02
	40-50%	1170 (10.0)	5 (6.7)	2.1	0.7-6.8	1.95E-01
	50-60%	1165 (10.0)	14 (18.7)	6.3	2.6-15.4	6.41E-05
	60-70%	1166 (10.0)	7 (9.3)	3.9	1.4-10.4	7.34E-03
	70-80%	1165 (10.0)	4 (5.3)	2.1	0.7-6.6	2.05E-01
	80-90%	1165 (10.0)	11 (14.7)	4.6	1.8-12.0	1.55E-03
	90-100%	1166 (10.0)	22 (29.3)	9.3	3.9-21.8	3.27E-07
breast cancer	0-30%	2476 (30.0)	6 (6.6)	1.0	reference	reference
	30-40%	826 (10.0)	3 (3.3)	1.7	0.5-5.8	3.96E-01
	40-50%	824 (10.0)	7 (7.7)	3.0	1.0-8.5	4.21E-02
	50-60%	826 (10.0)	9 (9.9)	3.9	1.4-10.5	7.15E-03
	60-70%	825 (10.0)	9 (9.9)	4.7	1.8-12.2	1.40E-03
	70-80%	826 (10.0)	16 (17.6)	6.8	2.8-16.5	2.63E-05
	80-90%	825 (10.0)	20 (22.0)	8.5	3.6-20.1	1.24E-06
	90-100%	826 (10.0)	21 (23.1)	8.9	3.8-21.0	6.31E-07
cervical cancer	0-30%	2476 (30.0)	7 (4.0)	1.0	reference	reference
	30-40%	826 (10.0)	9 (5.1)	3.7	1.5-9.5	5.64E-03
	40-50%	824 (10.0)	14 (7.9)	5.2	2.2-12.5	2.05E-04
	50-60%	826 (10.0)	24 (13.6)	9.1	4.1-20.2	7.86E-08
	60-70%	825 (10.0)	21 (11.9)	8.6	3.8-19.4	1.75E-07
	70-80%	826 (10.0)	31 (17.5)	11.5	5.3-25.2	8.59E-10
	80-90%	825 (10.0)	29 (16.4)	10.8	4.9-23.8	3.00E-09
	90-100%	826 (10.0)	42 (23.7)	15.6	7.3-33.4	1.40E-12

[a] OR, odds ratio

[b] CI, confidence interval

[c] *P* value and OR and 95% CI were calculated by univariate logistic model adjusted for age, and sex. All the tests were two-sided.

Supplementary Table 5 Sensitivity and specificity of ofCS-CD44 in different cancer stages

	sample size		AUC	AUC 95%CI	sensitivity	specificity
	case	control				
Pan-Cancer	1647	11654	0.73	0.71-0.74	0.66	0.68
Pan-Cancer-stage1	335	11654	0.65	0.62-0.67	0.83	0.39
Pan-Cancer-stage2	353	11654	0.72	0.69-0.74	0.59	0.74
Pan-Cancer-stage3	572	11654	0.75	0.73-0.77	0.70	0.68
Pan-Cancer-stage4	387	11654	0.77	0.74-0.79	0.79	0.64
bladder cancer	86	11654	0.81	0.77-0.85	0.85	0.66
bladder cancer-stage1	44	11654	0.77	0.71-0.83	0.73	0.69
bladder cancer-stage2	21	11654	0.85	0.80-0.91	0.9	0.76
bladder cancer-stage3	12	11654	0.82	0.73-0.90	0.92	0.71
bladder cancer-stage4	9	11654	0.87	0.80-0.93	1.00	0.66
esophageal squamous cell carcinoma	257	11654	0.79	0.76-0.81	0.85	0.66
esophageal squamous cell carcinoma-stage1	27	11654	0.76	0.68-0.85	0.73	0.69
esophageal squamous cell carcinoma-stage2	76	11654	0.78	0.74-0.82	0.9	0.76
esophageal squamous cell carcinoma-stage3	122	11654	0.79	0.75-0.82	0.92	0.71
esophageal squamous cell carcinoma-stage4	32	11654	0.84	0.78-0.90	1.00	0.66
gastric cancer	264	11654	0.75	0.72-0.78	0.69	0.72
gastric cancer-stage1	50	11654	0.68	0.61-0.75	0.56	0.72
gastric cancer-stage2	48	11654	0.8	0.75-0.86	0.85	0.68
gastric cancer-stage3	94	11654	0.76	0.72-0.81	0.79	0.66
gastric cancer-stage4	72	11654	0.75	0.69-0.80	0.65	0.76
nasopharyngeal carcinoma	223	11654	0.75	0.72-0.78	0.83	0.57
nasopharyngeal carcinoma-stage1	2	11654	0.49	0.00-1.00	0.50	0.78
nasopharyngeal carcinoma-stage2	13	11654	0.77	0.67-0.87	1.00	0.50
nasopharyngeal carcinoma-stage3	131	11654	0.75	0.71-0.79	0.84	0.58
nasopharyngeal carcinoma-stage4	77	11654	0.75	0.69-0.80	0.62	0.78
lung cancer	274	11654	0.75	0.73-0.78	0.65	0.72

Supplementary Table 5 Sensitivity and specificity of ofCS-CD44 in different cancer stages

	sample size		AUC	AUC 95%CI	sensitivity	specificity
	case	control				
lung cancer-stage1	71	11654	0.69	0.64-0.74	0.92	0.40
lung cancer-stage2	36	11654	0.73	0.65-0.81	0.61	0.82
lung cancer-stage3	76	11654	0.78	0.74-0.82	0.93	0.55
lung cancer-stage4	91	11654	0.78	0.74-0.83	0.69	0.77
colorectal cancer	200	11654	0.73	0.70-0.76	0.67	0.68
colorectal cancer-stage1	4	11654	0.65	0.47-0.82	1.00	0.45
colorectal cancer-stage2	43	11654	0.79	0.73-0.85	0.79	0.67
colorectal cancer-stage3	100	11654	0.70	0.65-0.74	0.75	0.55
colorectal cancer-stage4	53	11654	0.75	0.68-0.81	0.79	0.64
pancreatic cancer	75	11654	0.73	0.68-0.79	0.73	0.67
pancreatic cancer-stage1	3	11654	0.45	0.28-0.63	1.00	0.42
pancreatic cancer-stage2	10	11654	0.65	0.47-0.83	0.50	0.82
pancreatic cancer-stage3	19	11654	0.73	0.61-0.86	0.68	0.73
pancreatic cancer-stage4	43	11654	0.76	0.70-0.83	0.84	0.65
breast cancer	91	8254	0.70	0.65-0.75	0.74	0.43
breast cancer-stage1	30	8254	0.66	0.57-0.75	0.77	0.50
breast cancer-stage2	38	8254	0.70	0.63-0.77	0.79	0.61
breast cancer-stage3	14	8254	0.70	0.60-0.80	0.79	0.68
breast cancer-stage4	9	8254	0.83	0.68-0.97	0.78	0.83
cervical cancer	177	8254	0.69	0.65-0.72	0.92	0.25
cervical cancer-stage1	104	8254	0.68	0.63-0.72	0.80	0.49
cervical cancer-stage2	68	8254	0.70	0.65-0.75	0.88	0.45
cervical cancer-stage3	4	8254	0.72	0.38-1.00	0.75	0.74
cervical cancer-stage4	1	8254	0.90	NA-NA	1.00	0.90
