# Supplementary material for: Establishment and validation of a plasma oncofetal chondroitin sulfated proteoglycan for pan-cancer detection
Source: Nat Commun. 2023 Feb 6;14:645. doi: 10.1038/s41467-023-36374-7 (PMC9902466; doi:10.1038/s41467-023-36374-7)
Supplement: Supplementary file 2 — Reporting Summary [file 41467_2023_36374_MOESM2_ESM.pdf]

nature portfolio

Corresponding author(s): Wei-Hua Jia, Jianbing Mu

Last updated by author(s): Jan 13, 2023

Reporting Summary

Nature Portfolio wishes to improve the reproducibility of the work that we publish. This form provides structure for consistency and transparency in reporting. For further information on Nature Portfolio policies, see our Editorial Policies and the Editorial Policy Checklist.

Statistics

For all statistical analyses, confirm that the following items are present in the figure legend, table legend, main text, or Methods section.

n/a	Confirmed	
☐	☒	The exact sample size (n) for each experimental group/condition, given as a discrete number and unit of measurement
☐	☒	A statement on whether measurements were taken from distinct samples or whether the same sample was measured repeatedly
☐	☒	The statistical test(s) used AND whether they are one- or two-sided<br>*Only common tests should be described solely by name; describe more complex techniques in the Methods section.*
☐	☒	A description of all covariates tested
☐	☒	A description of any assumptions or corrections, such as tests of normality and adjustment for multiple comparisons
☐	☒	A full description of the statistical parameters including central tendency (e.g. means) or other basic estimates (e.g. regression coefficient) AND variation (e.g. standard deviation) or associated estimates of uncertainty (e.g. confidence intervals)
☐	☒	For null hypothesis testing, the test statistic (e.g. F, t, r) with confidence intervals, effect sizes, degrees of freedom and P value noted<br>*Give P values as exact values whenever suitable.*
☒	☐	For Bayesian analysis, information on the choice of priors and Markov chain Monte Carlo settings
☒	☐	For hierarchical and complex designs, identification of the appropriate level for tests and full reporting of outcomes
☐	☒	Estimates of effect sizes (e.g. Cohen's d, Pearson's r), indicating how they were calculated

Our web collection on statistics for biologists contains articles on many of the points above.

Software and code

Policy information about availability of computer code

Data collection	SPR was performed on a BIAcore T200 (GE healthcare, Uppsala, Sweden). Immunofluorescence images were taken by laser scanning confocal microscope (LSM 880, ZEISS). Flow cytometric was performed by cytoFLEX (2.2 Beckman). The optical density of Enzyme-Linkd Immunosorbent Assay was taken at 450 nm on microplate spectrophotometer (Epoch TM 2, Biotek)
Data analysis	(R-v 3.6.0), Zen Blue (v2.5, Zen blue edition, Zeiss, Germany) and GraphPad Prism (v 8.4.3)

For manuscripts utilizing custom algorithms or software that are central to the research but not yet described in published literature, software must be made available to editors and reviewers. We strongly encourage code deposition in a community repository (e.g. GitHub). See the Nature Portfolio guidelines for submitting code & software for further information.

Data

Policy information about availability of data

All manuscripts must include a data availability statement. This statement should provide the following information, where applicable:

- Accession codes, unique identifiers, or web links for publicly available datasets
- A description of any restrictions on data availability
- For clinical datasets or third party data, please ensure that the statement adheres to our policy

The ELISA data generated in this study have been deposited in the Figshare database with DOI: 10.6084/m9.figshare.21723716 [https://figshare.com]. The baseline patient information is provided in the Supplementary Information. The summary statistics that support the findings of this study are provided in the Supplementary

Information and Source Data file. Source data are provided with this paper.

Human research participants

Policy information about studies involving human research participants and Sex and Gender in Research.

Reporting on sex and gender	For cervical cancer and breast cancer, only female samples were used. Sex and gender were not considered in study design. Sex was determined based on self-reporting. Biological sex was included as a covariate in this study, the logistic regression model used for ROC analysis was developed and calculated as follows: logit (score) = α+β1×ofCS modified protein+β2×gender +β3×age.
Population characteristics	From Sun Yat-Sen University Cancer Center, a total of 165 patients (126 males and 39 females) who were diagnosed in 2019 were recruited as cases in the biomarker discovery set, and the age distribution were 55 (25-82) and 55 (27-75). In the validation cohort 1 study, 400 cancer samples (203 males and 197 females) diagnosed from 2015-2022 were collected from Tianjin Cancer Center and 21 regions in China other than Guangzhou, the age distribution were 60 (34-74) and 57 (27-85). In the validation cohort 2, a total of 2081 malignant tumor patients (1235 males and 846 females) who were diagnosed between Jan. 2013 to Dec. 2015 were used, and the age distribution were 55 (30-80) and 53 (30-80). For health controls, we randomly selected 302 healthy participants (121 males and 181 females) in the biomarker discovery set, 11654 healthy participants (3400 males and 8254 females) in the validation cohort, and 200 healthy participants (99 males and 101 females) in the external validation cohort. The age distribution were 44 (15-78) and 57 (16-76), 58 (16-94) and 51 (16-93), 51 (22-84) and 52 (19-86), respectively.
Recruitment	Malignant tumor patients were recruited from different cancer centers in China: the Sun Yat-Sen University Cancer Center (for the biomarker discovery and validation) and Tianjin Cancer Center (for external validation). In this study, the inclusion criteria for malignant tumor patients' specimens were as follows: (1) all subjects were histologically confirmed with malignant tumor, (2) the malignant tumor patients did not receive any clinical treatment such as chemotherapy or radiotherapy before blood collection or surgery.<br>Healthy subjects without evidence of any cancer were selected from Guangdong Biobank Cohort Study (ChiCTR1800015736) and the routine general health check-ups in both cancer centers.
Ethics oversight	This study was approved by the Ethics committees of SYSUCC and Tianjin cancer center (approval number: GZJZ-SB2016-022), and written informed consent was obtained from all participants to use their plasma samples. Two blood samples from Acute Lymphocytic Leukemia patients and a healthy donor were collected for FACS analysis.

Note that full information on the approval of the study protocol must also be provided in the manuscript.

Field-specific reporting

Please select the one below that is the best fit for your research. If you are not sure, read the appropriate sections before making your selection.

☒ Life sciences ☐ Behavioural & social sciences ☐ Ecological, evolutionary & environmental sciences

For a reference copy of the document with all sections, see nature.com/documents/nr-reporting-summary-flat.pdf

Life sciences study design

All studies must disclose on these points even when the disclosure is negative.

Sample size	We have run the power analysis with sample size calculation in the study design. We assumed the biomarker with ORs of 2, 3, and 5, and the case-control ratio of 1:10, 1:3, 1:1, and 2:1. We used the R package "sample size logistic case-control" to perform power and sample size analysis at the setting of the type one error of 0.05 and the disease prevalence of 100/100000. We found that with the most conservative estimation (OR=2 and case-control ratio=1:10), the total sample size required is about 250 to achieve a power of 0.9 . In the discovery stage, we selected a larger sample size of about 30 for each cancer type and a sample size of about 300 for the healthy control, which would have sufficient power. In order to validate the stability and consistent discrimination ability of the identified biomarker, we enlarged our sample size to a total of 400 cases and 200 controls (validation cohort 1), and 2081 cases and 11654 controls (validation cohort 2).
Data exclusions	no data were excluded from the analysis
Replication	We select 5% samples for replication, all attempts to replicate the experiments performed were successful. Other test in the article has been performed for 2-3 times independently with similar results.
Randomization	In this study, plasma samples from different malignancies and healthy controls were arranged in the same reaction plate for detection. That is, each sample was given a random number, and samples were randomly disrupted according to the size of the random number, and then arranged in the same test board for testing, so as to reduce the systematic error caused by the difference between boards and samples. For other experiments, allocation was not random because the analyses could be hardly influenced by investigator bias.
Blinding	For cell-based in vitro experiments, the investigators were not blinded on data acquasition and analysis.This was deemed unnecessary since no clinical trials were conducted in this study and none of the analyses reported involved procedures that could be influenced by investigator

bias. In the ELISA assay, the processing procedures were not blinding since we random the sample together to avoid the experiment bias.

Behavioural & social sciences study design

All studies must disclose on these points even when the disclosure is negative.

Study description	*Briefly describe the study type including whether data are quantitative, qualitative, or mixed-methods (e.g. qualitative cross-sectional, quantitative experimental, mixed-methods case study).*
Research sample	*State the research sample (e.g. Harvard university undergraduates, villagers in rural India) and provide relevant demographic information (e.g. age, sex) and indicate whether the sample is representative. Provide a rationale for the study sample chosen. For studies involving existing datasets, please describe the dataset and source.*
Sampling strategy	*Describe the sampling procedure (e.g. random, snowball, stratified, convenience). Describe the statistical methods that were used to predetermine sample size OR if no sample-size calculation was performed, describe how sample sizes were chosen and provide a rationale for why these sample sizes are sufficient. For qualitative data, please indicate whether data saturation was considered, and what criteria were used to decide that no further sampling was needed.*
Data collection	*Provide details about the data collection procedure, including the instruments or devices used to record the data (e.g. pen and paper, computer, eye tracker, video or audio equipment) whether anyone was present besides the participant(s) and the researcher, and whether the researcher was blind to experimental condition and/or the study hypothesis during data collection.*
Timing	*Indicate the start and stop dates of data collection. If there is a gap between collection periods, state the dates for each sample cohort.*
Data exclusions	*If no data were excluded from the analyses, state so OR if data were excluded, provide the exact number of exclusions and the rationale behind them, indicating whether exclusion criteria were pre-established.*
Non-participation	*State how many participants dropped out/declined participation and the reason(s) given OR provide response rate OR state that no participants dropped out/declined participation.*
Randomization	*If participants were not allocated into experimental groups, state so OR describe how participants were allocated to groups, and if allocation was not random, describe how covariates were controlled.*

Ecological, evolutionary & environmental sciences study design

All studies must disclose on these points even when the disclosure is negative.

Study description	*Briefly describe the study. For quantitative data include treatment factors and interactions, design structure (e.g. factorial, nested, hierarchical), nature and number of experimental units and replicates.*
Research sample	*Describe the research sample (e.g. a group of tagged Passer domesticus, all Stenocereus thurberi within Organ Pipe Cactus National Monument), and provide a rationale for the sample choice. When relevant, describe the organism taxa, source, sex, age range and any manipulations. State what population the sample is meant to represent when applicable. For studies involving existing datasets, describe the data and its source.*
Sampling strategy	*Note the sampling procedure. Describe the statistical methods that were used to predetermine sample size OR if no sample-size calculation was performed, describe how sample sizes were chosen and provide a rationale for why these sample sizes are sufficient.*
Data collection	*Describe the data collection procedure, including who recorded the data and how.*
Timing and spatial scale	*Indicate the start and stop dates of data collection, noting the frequency and periodicity of sampling and providing a rationale for these choices. If there is a gap between collection periods, state the dates for each sample cohort. Specify the spatial scale from which the data are taken*
Data exclusions	*If no data were excluded from the analyses, state so OR if data were excluded, describe the exclusions and the rationale behind them, indicating whether exclusion criteria were pre-established.*
Reproducibility	*Describe the measures taken to verify the reproducibility of experimental findings. For each experiment, note whether any attempts to repeat the experiment failed OR state that all attempts to repeat the experiment were successful.*
Randomization	*Describe how samples/organisms/participants were allocated into groups. If allocation was not random, describe how covariates were controlled. If this is not relevant to your study, explain why.*
Blinding	*Describe the extent of blinding used during data acquisition and analysis. If blinding was not possible, describe why OR explain why blinding was not relevant to your study.*

Did the study involve field work? ☐ Yes ☐ No

Field work, collection and transport

Field conditions	*Describe the study conditions for field work, providing relevant parameters (e.g. temperature, rainfall).*
Location	*State the location of the sampling or experiment, providing relevant parameters (e.g. latitude and longitude, elevation, water depth).*
Access & import/export	*Describe the efforts you have made to access habitats and to collect and import/export your samples in a responsible manner and in compliance with local, national and international laws, noting any permits that were obtained (give the name of the issuing authority, the date of issue, and any identifying information).*
Disturbance	*Describe any disturbance caused by the study and how it was minimized.*

Reporting for specific materials, systems and methods

We require information from authors about some types of materials, experimental systems and methods used in many studies. Here, indicate whether each material, system or method listed is relevant to your study. If you are not sure if a list item applies to your research, read the appropriate section before selecting a response.

Materials & experimental systems

n/a	Involved in the study	
☐	☒	Antibodies
☐	☒	Eukaryotic cell lines
☒	☐	Palaeontology and archaeology
☒	☐	Animals and other organisms
☒	☐	Clinical data
☒	☐	Dual use research of concern

Methods

n/a	Involved in the study	
☒	☐	ChIP-seq
☐	☒	Flow cytometry
☒	☐	MRI-based neuroimaging

Antibodies

Antibodies used	The antibodies used in Immunofluorescence staining and Flow Cytometry: FITC conjugated anti V5 antibody (R963-25, ThermoFisher), 1:500 dilution;<br>List of the antibodies used in Western blot : Anti-V5 Tag Antibody(ab53418, abcam 1:5000 dilution); Goat anti-mouse IgG+IgM H&L (HRP)(ab47827, Abcam 1:5000 dilution);<br>List of the antibodies used in ELISA and immunofluorescence stainning assay: anti-CD44 (Proteintech, Cat#60224-1-Ig), -CSPG4 (Proteintech, Cat#55027-1-AP), and -SDC1 (Proteintech, Cat#67155-1-Ig) , anti-rabbit IgG (H+L), F(ab')2 Fragment (Cell Signaling, Cat#4413 or 4409) , FITC conjugated anti V5 antibody (R963-25, ThermoFisher)
Validation	All primary antibodies are commercially available. Antibody validation was provided by the company. Information of antibody validation is provided on manufacturer's official website, which shows validated data from multiple species of cells for the experiment of WB, IF, IP, IHC, and so on. Validation data can be accessed by searching for the listed catalog numbers on the Abcam (http://www.abcam.cn/), ThermoFisher (https://www.thermofisher.com/), Proteintech (https://www.ptgcn.com/) , Cell Signaling (https://www.cellsignal.cn/) websited.

Eukaryotic cell lines

Policy information about cell lines and Sex and Gender in Research

Cell line source(s)	A panel of cell lines used in this study, A549, SW480, SW620, HT29, Caco2, LOVO, and HCT116 were purchased from commercial companies (CellCook, https://www.cellcook.com/) and stored in our lab. ESCC cell lines (KYSE30 and KYSE180) were generously provided by Professor Guan from SYSUCC. Sf9 were generously provided by Professor Gao from SYSUCC.
Authentication	All cell lines have been validated by the supplier through genetic profiling.
Mycoplasma contamination	Mycoplasma contamination was not detected in the cell lines acquired.
Commonly misidentified lines (See ICLAC register)	Not commonly misidentified cell lines were used.

Palaeontology and Archaeology

Specimen provenance	*Provide provenance information for specimens and describe permits that were obtained for the work (including the name of the issuing authority, the date of issue, and any identifying information). Permits should encompass collection and, where applicable, export.*
Specimen deposition	*Indicate where the specimens have been deposited to permit free access by other researchers.*
Dating methods	*If new dates are provided, describe how they were obtained (e.g. collection, storage, sample pretreatment and measurement), where they were obtained (i.e. lab name), the calibration program and the protocol for quality assurance OR state that no new dates are provided.*

☐ Tick this box to confirm that the raw and calibrated dates are available in the paper or in Supplementary Information.

Ethics oversight	*Identify the organization(s) that approved or provided guidance on the study protocol, OR state that no ethical approval or guidance was required and explain why not.*

Note that full information on the approval of the study protocol must also be provided in the manuscript.

Animals and other research organisms

Policy information about studies involving animals; ARRIVE guidelines recommended for reporting animal research, and Sex and Gender in Research

Laboratory animals	*For laboratory animals, report species, strain and age OR state that the study did not involve laboratory animals.*
Wild animals	*Provide details on animals observed in or captured in the field; report species and age where possible. Describe how animals were caught and transported and what happened to captive animals after the study (if killed, explain why and describe method; if released, say where and when) OR state that the study did not involve wild animals.*
Reporting on sex	*Indicate if findings apply to only one sex; describe whether sex was considered in study design, methods used for assigning sex. Provide data disaggregated for sex where this information has been collected in the source data as appropriate; provide overall numbers in this Reporting Summary. Please state if this information has not been collected. Report sex-based analyses where performed, justify reasons for lack of sex-based analysis.*
Field-collected samples	*For laboratory work with field-collected samples, describe all relevant parameters such as housing, maintenance, temperature, photoperiod and end-of-experiment protocol OR state that the study did not involve samples collected from the field.*
Ethics oversight	*Identify the organization(s) that approved or provided guidance on the study protocol, OR state that no ethical approval or guidance was required and explain why not.*

Note that full information on the approval of the study protocol must also be provided in the manuscript.

Clinical data

Policy information about clinical studies

All manuscripts should comply with the ICMJEguidelines for publication of clinical research and a completedCONSORT checklist must be included with all submissions.

Clinical trial registration	*Provide the trial registration number from ClinicalTrials.gov or an equivalent agency.*
Study protocol	*Note where the full trial protocol can be accessed OR if not available, explain why.*
Data collection	*Describe the settings and locales of data collection, noting the time periods of recruitment and data collection.*
Outcomes	*Describe how you pre-defined primary and secondary outcome measures and how you assessed these measures.*

Dual use research of concern

Policy information about dual use research of concern

Hazards

Could the accidental, deliberate or reckless misuse of agents or technologies generated in the work, or the application of information presented in the manuscript, pose a threat to:

No	Yes	
☐	☐	Public health
☐	☐	National security
☐	☐	Crops and/or livestock
☐	☐	Ecosystems
☐	☐	Any other significant area

Experiments of concern

Does the work involve any of these experiments of concern:

No	Yes	
☐	☐	Demonstrate how to render a vaccine ineffective
☐	☐	Confer resistance to therapeutically useful antibiotics or antiviral agents
☐	☐	Enhance the virulence of a pathogen or render a nonpathogen virulent
☐	☐	Increase transmissibility of a pathogen
☐	☐	Alter the host range of a pathogen
☐	☐	Enable evasion of diagnostic/detection modalities
☐	☐	Enable the weaponization of a biological agent or toxin
☐	☐	Any other potentially harmful combination of experiments and agents

ChIP-seq

Data deposition

☐ Confirm that both raw and final processed data have been deposited in a public database such as GEO.

☐ Confirm that you have deposited or provided access to graph files (e.g. BED files) for the called peaks.

Data access links<br>*May remain private before publication.*	*For "Initial submission" or "Revised version" documents, provide reviewer access links. For your "Final submission" document, provide a link to the deposited data.*
Files in database submission	*Provide a list of all files available in the database submission.*
Genome browser session<br>(e.g. UCSC)	*Provide a link to an anonymized genome browser session for "Initial submission" and "Revised version" documents only, to enable peer review. Write "no longer applicable" for "Final submission" documents.*

Methodology

Replicates	*Describe the experimental replicates, specifying number, type and replicate agreement.*
Sequencing depth	*Describe the sequencing depth for each experiment, providing the total number of reads, uniquely mapped reads, length of reads and whether they were paired- or single-end.*
Antibodies	*Describe the antibodies used for the ChIP-seq experiments; as applicable, provide supplier name, catalog number, clone name, and lot number.*
Peak calling parameters	*Specify the command line program and parameters used for read mapping and peak calling, including the ChIP, control and index files used.*
Data quality	*Describe the methods used to ensure data quality in full detail, including how many peaks are at FDR 5% and above 5-fold enrichment.*
Software	*Describe the software used to collect and analyze the ChIP-seq data. For custom code that has been deposited into a community repository, provide accession details.*

Flow Cytometry

Plots

Confirm that:

- [x] The axis labels state the marker and fluorochrome used (e.g. CD4-FITC).
- [x] The axis scales are clearly visible. Include numbers along axes only for bottom left plot of group (a 'group' is an analysis of identical markers).
- [x] All plots are contour plots with outliers or pseudocolor plots.
- [x] A numerical value for number of cells or percentage (with statistics) is provided.

Methodology

Sample preparation	The culture cells were dissociated by Gentle cell dissociation reagent (07174, Stem cell) at room temperature and washed with PBS by centrifugation at 450 ×g for 5 min. After blocking with 10% goat serum for 1 hour at room temperature using end-over-end rotation, the cells were incubated with rVAR2 (200nM to 25nM, a serial of 2-fold dilution) at room temperature for 1 hour, and 5% BSA was used as the negative control. The cells were incubated with FITC conjugated anti-V5 antibody (1:500, R963-25, ThermoFisher) and suspended in 5% BSA before the binding of rVAR2 was detected.
Instrument	CytoFLEX (Beckman)
Software	CytExpert 2.2.0.97
Cell population abundance	50,000 cells were analyzed for each sample.
Gating strategy	The initial cell population was generated by Forward Scatter and Slide Scatter to make sure double exclusion. Only single cell was used for analysis. In FSC/SSC plot, cells were distributed diagonally and two distinct cell populations can be identified. The bottom left one was considered as cell debris, and the other one was the starting cell population.

- [x] Tick this box to confirm that a figure exemplifying the gating strategy is provided in the Supplementary Information.

Magnetic resonance imaging

Experimental design

Design type	*Indicate task or resting state; event-related or block design.*
Design specifications	*Specify the number of blocks, trials or experimental units per session and/or subject, and specify the length of each trial or block (if trials are blocked) and interval between trials.*
Behavioral performance measures	*State number and/or type of variables recorded (e.g. correct button press, response time) and what statistics were used to establish that the subjects were performing the task as expected (e.g. mean, range, and/or standard deviation across subjects).*

Acquisition

Imaging type(s)	*Specify: functional, structural, diffusion, perfusion.*
Field strength	*Specify in Tesla*
Sequence & imaging parameters	*Specify the pulse sequence type (gradient echo, spin echo, etc.), imaging type (EPI, spiral, etc.), field of view, matrix size, slice thickness, orientation and TE/TR/flip angle.*
Area of acquisition	*State whether a whole brain scan was used OR define the area of acquisition, describing how the region was determined.*

Diffusion MRI ☐ Used ☐ Not used

Preprocessing

Preprocessing software	*Provide detail on software version and revision number and on specific parameters (model/functions, brain extraction, segmentation, smoothing kernel size, etc.).*
Normalization	*If data were normalized/standardized, describe the approach(es): specify linear or non-linear and define image types used for transformation OR indicate that data were not normalized and explain rationale for lack of normalization.*
Normalization template	*Describe the template used for normalization/transformation, specifying subject space or group standardized space (e.g. original Talairach, MNI305, ICBM152) OR indicate that the data were not normalized.*

Noise and artifact removal	*Describe your procedure(s) for artifact and structured noise removal, specifying motion parameters, tissue signals and physiological signals (heart rate, respiration).*
Volume censoring	*Define your software and/or method and criteria for volume censoring, and state the extent of such censoring.*

Statistical modeling & inference

Model type and settings	*Specify type (mass univariate, multivariate, RSA, predictive, etc.) and describe essential details of the model at the first and second levels (e.g. fixed, random or mixed effects; drift or auto-correlation).*
Effect(s) tested	*Define precise effect in terms of the task or stimulus conditions instead of psychological concepts and indicate whether ANOVA or factorial designs were used.*

Specify type of analysis: ☐ Whole brain ☐ ROI-based ☐ Both

Statistic type for inference<br>(See Eklund et al. 2016)	*Specify voxel-wise or cluster-wise and report all relevant parameters for cluster-wise methods.*
Correction	*Describe the type of correction and how it is obtained for multiple comparisons (e.g. FWE, FDR, permutation or Monte Carlo).*

Models & analysis

n/a	Involved in the study
☐	☐ Functional and/or effective connectivity
☐	☐ Graph analysis
☐	☐ Multivariate modeling or predictive analysis

Functional and/or effective connectivity	*Report the measures of dependence used and the model details (e.g. Pearson correlation, partial correlation, mutual information).*
Graph analysis	*Report the dependent variable and connectivity measure, specifying weighted graph or binarized graph, subject- or group-level, and the global and/or node summaries used (e.g. clustering coefficient, efficiency, etc.).*
Multivariate modeling and predictive analysis	*Specify independent variables, features extraction and dimension reduction, model, training and evaluation metrics.*
